# Investigation of habenula volume in mood disorders: A meta-analytic study

**DOI:** 10.1017/S0033291726103730

**Published:** 2026-03-30

**Authors:** Jean-Simon Fortin, Sébastien Hétu, Charles Parisien, Catherine Parent

**Affiliations:** Department of Psychology, Université de Montréal, Montreal, QC, Canada

**Keywords:** bipolar disorder, depression, habenula, meta-analysis, mood disorders, volume, MRI, atrophy, neurotrophic theory of depression

## Abstract

The habenula, a small brain structure involved in processing aversive stimuli, has been strongly implicated in the pathophysiology of mood disorders. While diminutions in hippocampal and medial prefrontal cortex volume have been demonstrated in individuals with a mood disorder, evidence for structural alterations in the habenula remains inconsistent. This set of meta-analyses examines whether individuals with a mood disorder show alterations in habenula volume compared to healthy controls. We conducted six meta-analyses. Two global analyses compared left and right habenula volumes between individuals with a mood disorder (MDD or BD) and healthy controls (HCs), each including 15 samples (left: 1,230 participants; right: 1,236). Four additional analyses compared MDD versus HCs and BD versus HCs for left and right volumes separately. Subgroup and meta-regression analyses tested the habenula segmentation method, medication status, and MRI resolution as moderators. The global meta-analyses pooling MDD and BD data showed small but significant volume reductions in the left (*g* = −0.1367, *p* = .0344) and right (*g* = −0.1562, *p* = .0409) habenula in mood disorder patients compared to controls. However, these effects did not survive correction for multiple comparisons. After correction, no significant group differences were found in the diagnosis-specific meta-analyses (MDD versus controls; BD versus controls), and no moderator analyses were significant. Current evidence points toward small habenula volume reductions in mood disorders, though findings did not withstand correction for multiple comparisons. Further high-resolution neuroimaging studies are needed to clarify habenula volume alterations in mood disorders.

Mood disorders – including major depressive disorder (MDD) and bipolar disorder (BD) – collectively rank among the leading contributors to the global burden of disease. Within the category of mental disorders, they account for the highest proportion of disability-adjusted life years (DALYs), a metric that captures both premature mortality and years lived with disability (GBD, 2019 Mental Disorders Collaborators, 2022). Worldwide, MDD affects an estimated 280 million people, and BD about 40 million (World Health Organization, 2022). Despite their impact, their underlying pathophysiology remains poorly understood (Jesulola, Micalos, & Baguley, 2018; Marcolongo-Pereira et al., 2022). In recent years, the habenula – a pair of small epithalamic nuclei located in the dorsal thalamus – has emerged as one of the most intensively studied brain structures in preclinical research on mood disorders (Spreen, Alkhoury, Walter, & Müller, 2024), and is receiving increasing attention in human studies (Cameron, Weston-Green, & Newell, 2024; Chen, Sun, Zhang, Mu, & Su, 2022). As a key node within the brain’s reward circuitry (Namboodiri, Rodriguez-Romaguera, & Stuber, 2016), the habenula plays a crucial role in processing aversive stimuli and is often referred to as the brain’s ‘anti-reward center’ (Hu, Cui, & Yang, 2020). It comprises two distinct subdivisions: the medial habenula (MHb) and the lateral habenula (LHb), with the LHb believed to account for a substantially greater proportion of total habenula volume in humans (Ahumada-Galleguillos et al., 2017; Groos & Helmchen, 2024).

The growing interest in the habenula’s role in mood disorders stems from animal studies implicating it as a key hub in their pathophysiology (Hikosaka, 2010; Hu et al., 2020; Yang, Wang, Hu, & Hu, 2018). Notably, one of the most influential discoveries has been the identification of elevated baseline activity in the LHb – a phenomenon consistently observed across diverse animal models of depression (Caldecott-Hazard, Mazziotta, & Phelps, 1988; Cerniauskas et al., 2019; Tchenio, Lecca, Valentinova, & Mameli, 2017) – which appears to play a critical role in the expression of depressive-like behaviors. Caldecott-Hazard et al. (1988) first reported elevated LHb glucose metabolism in three rodent depression models, identifying it as the only brain region consistently hyperactive in all three models. Since then, multiple lines of evidence have supported LHb hyperactivity as a key contributor to depression pathophysiology. For example, optogenetic stimulation of LHb projections to the rostromedial tegmental nucleus (RMTg) was shown to induce depressive-like symptoms in rodents (Proulx et al., 2018), while LHb inhibition produced antidepressant-like effects (Winter, Vollmayr, Djodari-Irani, Klein, & Sartorius, 2011). Additional studies have shown that lesioning the LHb reduces depressive-like behaviors following chronic stress (Yang, Hu, Xia, Zhang, & Zhao, 2008) or prevents their emergence when performed prior to exposure to inescapable shocks (Amat et al., 2001). Furthermore, the rapid antidepressant effects of ketamine have been linked to its ability to normalize the increased burst firing of LHb neurons observed in animal models of depression (Yang et al., 2018). Collectively, these findings strongly implicate excessive LHb activity as a central mechanism in the pathophysiology of depression. Given the habenula’s well-documented functional abnormalities in mood disorders, it is plausible that it may also exhibit structural alterations. This notion is supported by animal studies showing reduced habenula volume in depression models (Jacinto, Mata, Novais, Marques, & Sousa, 2017; Yang et al., 2019), as well as longitudinal human studies reporting volume increases following treatments such as antidepressants (Elias et al., 2022; Etienne et al., 2024; Sartorius et al., 2016). Such findings have spurred growing interest in habenula anatomy, reflected in a growing number of studies examining habenula volume in individuals with a mood disorder (Cameron et al., 2024).

More generally, human structural neuroimaging has been instrumental in advancing our understanding of mood disorders. Among the most consistently observed brain alterations in MDD are reductions in hippocampal and medial prefrontal cortex (mPFC) volume (Belleau, Treadway, & Pizzagalli, 2019; Campbell, Marriott, Nahmias, & MacQueen, 2004; Koolschijn, van Haren, Lensvelt-Mulders, Hulshoff Pol, & Kahn, 2009; Schmaal et al., 2016), with similar reductions reported in BD (Angelescu, Brugger, Borgan, Kaar, & Howes, 2021; Cao et al., 2017; Hajek, Kopecek, Höschl, & Alda, 2012; Haukvik et al., 2022; Qi et al., 2022; Savitz, Price, & Drevets, 2014; Wise et al., 2017). Findings of volume reductions in these stress-sensitive brain regions have been instrumental in the development of the neurotrophic hypothesis of depression, a leading alternative to the monoaminergic theory (Ferrari & Villa, 2017; Liu, Liu, Wang, Zhang, & Li, 2017; Page, Epperson, Novick, Duffy, & Thompson, 2024). According to this hypothesis, stress reduces the expression of neurotrophic factors – particularly brain-derived neurotrophic factor (BDNF) – in key limbic regions. This reduction in BDNF is thought to drive structural degeneration, most notably in the hippocampus and mPFC (Duman & Monteggia, 2006). In both regions, structural abnormalities co-occur with well-documented functional impairments (Liu et al., 2017; Page et al., 2024). Recent evidence shows that stress also reduces BDNF levels in the habenula (Sachs & Caron, 2015; Tong et al., 2023; Zhang et al., 2022) – a change that, as in the hippocampus and mPFC (Björkholm & Monteggia, 2016; Duman, Deyama, & Fogaça, 2021), can be reversed by antidepressant treatment. These findings raise the possibility that neurotrophic mechanisms contributing to structural alterations in the hippocampus and mPFC may likewise affect the habenula, another key region involved in mood regulation. Such mechanisms could potentially lead to volume reductions in the habenula in mood disorders.

Evidence of volumetric alterations in the habenula could have wide-ranging implications, potentially prompting research aimed at integrating the habenula into the neurotrophic theory of depression and at exploring its relationship with the hypothalamic–pituitary–adrenal (HPA) axis. Moreover, these findings could establish the habenula as a crucial node in research seeking to bridge reward-based and neurotrophic models of depression, aiming to provide a more unified framework for understanding its pathophysiology. Existing studies on habenula volume in mood disorders show mixed results: some report reduced habenula volume in mood disorders (Cho et al., 2021; Ranft et al., 2010), while others observe no differences (Aftanas et al., 2023; Etienne et al., 2023; Hou et al., 2025; Lawson et al., 2017) or even increased volume (Liu, Valton, Wang, Zhu, & Roiser, 2017). Therefore, to clarify whether habenula volume is reliably altered in mood disorders, we conducted six meta-analyses comparing patients and healthy controls (HCs). Specifically, we examined left and right habenula volume separately, comparing individuals with MDD and BD – both pooled and as distinct groups – to HCs. Given the habenula’s small size (~30 mm^3^), current imaging lacks the resolution to distinguish its lateral and medial subdivisions; therefore, our meta-analyses focused on total habenula volume.

## Methods and materials

This meta-analysis followed the PRISMA guidelines (Page et al., 2021) and was registered on OSF (https://osf.io/atxvj/).

### Literature search

We searched Ovid (Embase, Medline, PsycINFO), Web of Science, and Google Scholar in April 2025 (see Supplemental Information for full strategies).

### Inclusion and exclusion criteria

To be included, studies had to report habenula volumes (MRI or post-mortem) in humans and include: (1) a mood disorder group (MDD or BD), (2) a HC group, and (3) separate data for left and right habenula. Only studies reporting whole habenula volume were included; those with only gray/white matter measures were excluded. Left and right volumes were analyzed separately based on evidence of lateralized habenula function (Concha & Ahumada-Galleguillos, 2016; Hétu et al., 2016). Studies were eligible regardless of publication date or format (e.g. preprints, theses, conference proceedings). Non-English articles and conference abstracts were excluded, though authors would have been contacted to obtain the data if conference abstracts seemed to be relevant (none identified). Only studies on MDD or BD were included; those on other conditions (e.g. PTSD) or subclinical depression (e.g. elevated symptoms without a clinical diagnosis) were excluded.

### Study selection

**Figure 1.**
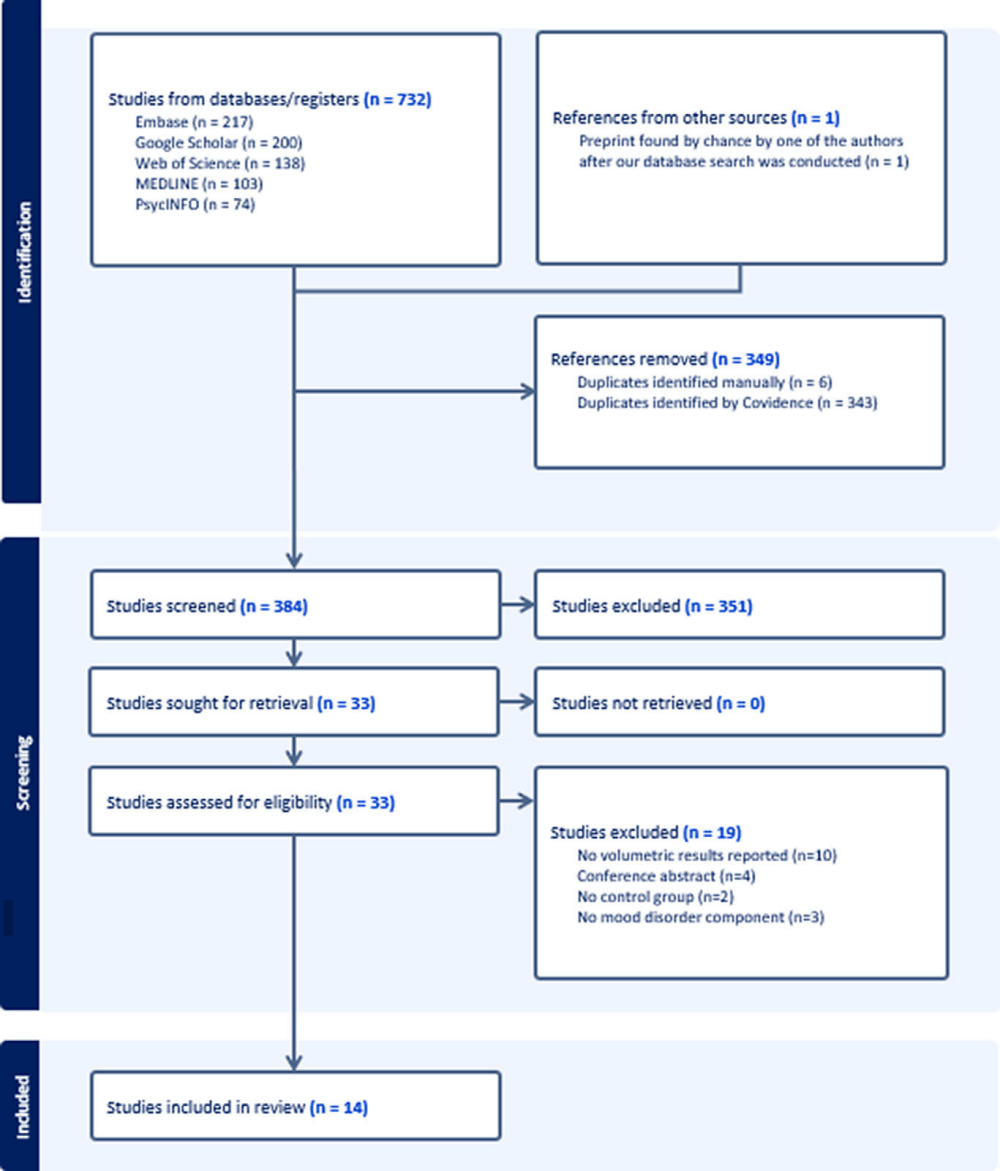
PRISMA flow diagram of the study selection process.

The study selection process is shown in the PRISMA diagram (Figure 1) (Covidence systematic review software, Veritas Health Innovation, Melbourne, Australia. Available at www.covidence.org.). One reviewer (J.S.F) conducted the initial database search and imported results into Covidence, where both title/abstract and full-text screening were performed. Two reviewers (J.S.F and C.P2) independently screened all titles/abstracts and full texts, resolving discrepancies through discussion. The initial search yielded 732 studies across five databases, with one additional preprint identified independently after the search. Duplicates were removed using Covidence’s automated tool and manual checks, leaving 384 unique records. Of these, 33 were assessed at the full-text level. In total, 14 studies (15 samples) met the inclusion criteria and were incorporated into the global meta-analyses, pooling data across both MDD and BD.

### Data extraction

We extracted data required for effect size calculations: sample size, mean, and standard deviation of left and right habenula volumes for clinical and control groups. Two reviewers (J.S.F and C.P2) independently extracted these values for all datasets and cross-checked results to resolve discrepancies. Data were recorded in Excel. We used absolute habenula volumes (in mm^3^) rather than relative measures (e.g. based on total intracranial volume (TIV) or total brain volume (TBV)), as absolute values were more consistently reported across studies.

### Quality assessment

Study quality was assessed by one reviewer (J.S.F) using the NIH tool for observational studies (National Heart Lung and Blood Institute (NHLBI), 2021).

### Data analysis

We conducted six meta-analyses. First, we performed two meta-analyses comparing left and right habenula volumes between individuals with a mood disorder (MDD or BD) and HCs; these are referred to as the global meta-analyses throughout the paper. Two additional meta-analyses focused on MDD, and two on BD, each comparing patients to controls for left and right volumes. To control for multiple comparisons across our six meta-analyses, a 5% false discovery rate (FDR) correction (Benjamini–Hochberg procedure (Benjamini & Hochberg, 1995) was applied. Unless otherwise noted, *p*-values reported in the article are uncorrected. A random-effects model with inverse-variance weighting was used, and Hedges’ g was chosen as the effect size metric to correct for small-sample bias (Hedges & Olkin, 1985).

We conducted secondary subgroup analyses by habenula segmentation method and medication status within each of the six meta-analyses. Automatic and semi-automatic habenula segmentation methods were combined and compared to manual segmentation. Groups were classified as unmedicated only if none of the participants used psychotropic medication at the time of scanning; otherwise, they were considered medicated. Prior medication history did not affect classification. Sex-based subgroup analyses were not conducted, as only one study reported habenula volumes by sex. We performed subgroup analyses in all meta-analyses regardless of the number of samples. However, subgroup comparisons with fewer than 10 studies (*k* < 10) should be interpreted cautiously due to low power and greater uncertainty in effect size estimates. When *k* ≥ 10, meta-regressions tested MRI resolution (mean-centered in mm^3^) as a moderator. A 5% FDR correction controlled for multiple comparisons across all secondary analyses.

## Results

### Sample characteristics

Fourteen articles (15 samples, as one study contained two samples) were included in the global meta-analyses pooling MDD and BD. Detailed sample characteristics are reported in Table 1. Fourteen samples used structural MRI, and one used post-mortem data (Ranft et al., 2010). Among MRI studies, two used 7 T scanners (Cho et al., 2021; Schmidt et al., 2017), ten used 3 T (Aftanas et al., 2023; Etienne et al., 2023; Furman & Gotlib, 2016; Hou et al., 2025; Kyuragi et al., 2024; Lawson et al., 2017; Liu, Valton, et al, 2017; Luan, Zhang, Wang, Zhao, & Liu, 2019; Savitz et al., 2011; Schafer et al., 2018), and two used 1.5 T (Germann et al., 2020). Manual habenula segmentation was applied in nine MRI samples (Aftanas et al., 2023; Cho et al., 2021; Etienne et al., 2023; Furman & Gotlib, 2016; Hou et al., 2025; Lawson et al., 2017; Liu, Valton, et al., 2017; Luan et al., 2019; Savitz et al., 2011) and one post-mortem sample (Ranft et al., 2010). Three samples used fully automated segmentation (Germann et al., 2020; Kyuragi et al., 2024, and two used semi-automated methods (Schafer et al., 2018; Schmidt et al., 2017). MRI voxel resolution ranged from 0.125 to 2.028 mm^3^ (mean = 0.591 ± 0.505 mm^3^). Four samples did not report medication status (Germann et al., 2020; Kyuragi et al., 2024; Luan et al., 2019). In four other samples, all patients were medication-free at the time of scanning (Etienne et al., 2023; Hou et al., 2025; Lawson et al., 2017; Liu, Valton, et al., 2017). Of these, one study reported that 27 of 64 MDD participants had prior antidepressant use (Etienne et al., 2023); others did not report treatment history (Hou et al., 2025; Lawson et al., 2017; Liu, Valton, et al., 2017). One study provided separate data for medicated and unmedicated MDD patients (Schmidt et al., 2017), and six samples included some medicated participants (Aftanas et al., 2023; Cho et al., 2021; Furman & Gotlib, 2016; Ranft et al., 2010; Savitz et al., 2011; Schafer et al., 2018). Based on the NIH Quality Assessment Tool, three samples were rated good, ten fair, and two poor (Supplementary Table S1).

**Table 1.** Summary of characteristics of included samples

Authors	Clinical features of study groups	Number of participants in the group (female)	Age (*M*, *SD*)	Medication status	Method	Resolution for image acquisition	Magnet strength	Segmentation method for the habenula	Country
Aftanas et al. (2023)	HC	41 (28)	41.8 ± 9.0	NA	Volumetric measures using structural MRI	0.5 × 0.5 × 0.5 mm	3 T	Manual segmentation was conducted for each participant individually based on Lawson et al.	Russia
	MDD	84 (56)	43.2 ± 12.2	23 patients were taking antidepressants, while 61 were not.					
Cho et al. (2021)	HC	36 (24)	35.17 ± 12.00	NA	Volumetric measures using structural MRI	0.65 × 0.65 × 0.65 mm	7 T	Manual segmentation was conducted for each participant individually.	South Korea
	MDD	33 (25)	40.58 ± 14.16	27 patients were taking antidepressants, 19 benzodiazepines, and 14 second-generation antipsychotics.					
Etienne et al. (2023)	HC	32 (21)	35.13 ± 12.90		Volumetric measures using structural MRI	0.795 × 0.795 × 1.0 mm	3 T	Manual segmentation was conducted for each participant individually using BrainVISA imaging software (version 4.6) and the Anatomist module (version 4.6.1).	France
	MDD	64 (44)	34.25 ± 12.45	All patients were antidepressant-free at the time of the study, but 27 patients had a history of taking antidepressants					
Furman and Gotlib (2016)	HC	13	27.3 ± 7.0	NA	Volumetric measures using structural MRI	0.9 × 0.9 × 0.9 mm	3 T	Manual segmentation was conducted for each participant individually based on Lawson et al.	United States
	MDD	15	31.1 ± 6.9	5 patients were medicated while 10 patients were not. Among the 5 medicated patients, 4 were taking antidepressants, and one was taking a benzodiazepine					
Germann et al. (2020) CANDI sample^a^	HC	25 for the left habenula and 26 for the right habenula	NA	NA	Volumetric measures using structural MRI	0.93 × 0.93 × 1.5 mm	1.5 T	The habenula was identified using a fully automated algorithm proposed in the paper.	United States
	BD	50 for the left habenula and 49 for the right habenula	NA	NA					
Germann et al. (2020) Adult-BD sample^a^	HC	54 for the left habenula and 56 for the right habenula	NA	NA	Volumetric measures using structural MRI	1.2 × 1.3 × 1.3 mm	1.5 T	The habenula was identified using a fully automated algorithm proposed in the paper.	Brazil
	BD	28 for the left habenula and 32 for the right habenula	NA	NA					
Hou et al. (2025)	HC	47 (27)	35.68 ± 2.05	NA	Volumetric measures using structural MRI	0.8 × 0.8 × 0.8 mm	3 T	Manual segmentation was conducted for each participant individually.	China
	FED	47 (31)	30.26 ± 1.25	All patients were unmedicated					
Kyuragi et al. (2024)	HC	92	NA	NA	Volumetric measures using structural MRI	0.8 × 0.8 × 0.8 mm	3 T	Automatic habenula segmentation using a method developed in the paper.	Japan
	MDD	153	NA	NA					
Lawson et al. (2017)	HC	25 (11)	27.44 ± 8.75	NA	Volumetric measures using structural MRI	0.77 × 0.77 × 0.77 mm	3 T	Manual segmentation was conducted for each participant individually based on Lawson et al.	United Kingdom
	MDD	25 (10)	27.76 ± 9.01	All patients were unmedicated.					
Liu et al. (2017)	HC	17 (10)	28.3 ± 5.2	NA	Volumetric measures using structural MRI	0.75 × 0.75 × 0.75 mm	3 T	Manual segmentation was conducted for each participant individually based on Lawson et al.	China
	MDD	21 (12)	30.7 ± 8.9	All patients were unmedicated.					
Luan et al. (2019)	HC	15 (8)	33.5 ± 6.8	NA	Volumetric measures using structural MRI	0.5 × 0.5 × 1 mm	3 T	Manual segmentation was conducted for each participant individually.	China
	TRD	15 (6)	34.4 ± 6.2	NA					
Ranft et al. (2010)	HC	13 (5)	55.9 ± 8.9	NA	Volumetric measures using a computerized image analysis system and a Leica microscope. This study used post-mortem brains.	See the paper for details.	NA	Manual segmentation was conducted for each participant individually. However, this is not the usual MRI segmentation.	Germany
	MDD/BD (six patients with MDD and eight with BD)	14 (8)	48.6 ± 12.8	This was a post-mortem study. However, before death, all MDD patients had been taking antidepressants. All BD patients except one had been taking lithium.					
Savitz et al. (2011)	HC	74 (45)	37.1 ± 11.9	NA	Volumetric measures using structural MRI	0.55 × 0.55 × 0.6 mm	3 T	Manual segmentation was conducted for each participant individually.	United States
	MDD	28 (15)	43.9 ± 11.5	All patients were either medication-naive or had been unmedicated for at least 4 weeks.					
	Unmedicated BD	22 (15)	34.4 ± 6.7	All patients had been unmedicated for at least 2 months.					
	Medicated BD	15 (10)	45.1 ± 9.1	Eight patients were taking lithium, six divalproex, and one chlorpromazine.					
Schafer et al. (2018)	HC	40 (16)	29.78 ± 8.65	NA	Volumetric measures using structural MRI	0.8 × 0.8 × 0.8 mm	3 T	The habenula was identified using a semi-automated algorithm developed by Kim et al. (2016)	United States
	BD	32 (12)	28.19 ± 8.74	27 patients were taking at least one antipsychotic (6 were taking first-generation and 27 were taking second-generation antipsychotics), 8 antidepressants, 15 lithium, 6 anti-epileptics, and 1 was unmedicated.					
Schmidt et al. (2017)	HC	20 (12)	36.45 ± 13.16	NA	Volumetric measures using structural MRI	0.7 × 0.7 × 0.7 mm	7 T	The habenula was identified using a computer-assisted interactive segmentation algorithm developed in the paper.	Germany
	Unmedicated MDD	20 (13)	36.20 ± 12.83	All patients were unmedicated.					
	Medicated MDD	20 (12)	40.60 ± 12.11	All patients were medicated.					

*Note:* BD, bipolar disorder; FED, first-episode depression; HC, healthy control; MDD, major depressive disorder; MRI, magnetic resonance imaging; NA, not available; TRD, treatment-resistant depression.

a The sample size reported corresponds to the number of participants that could be accurately extracted from the available graphical data.

### Meta-analyses

**Figure 2.**
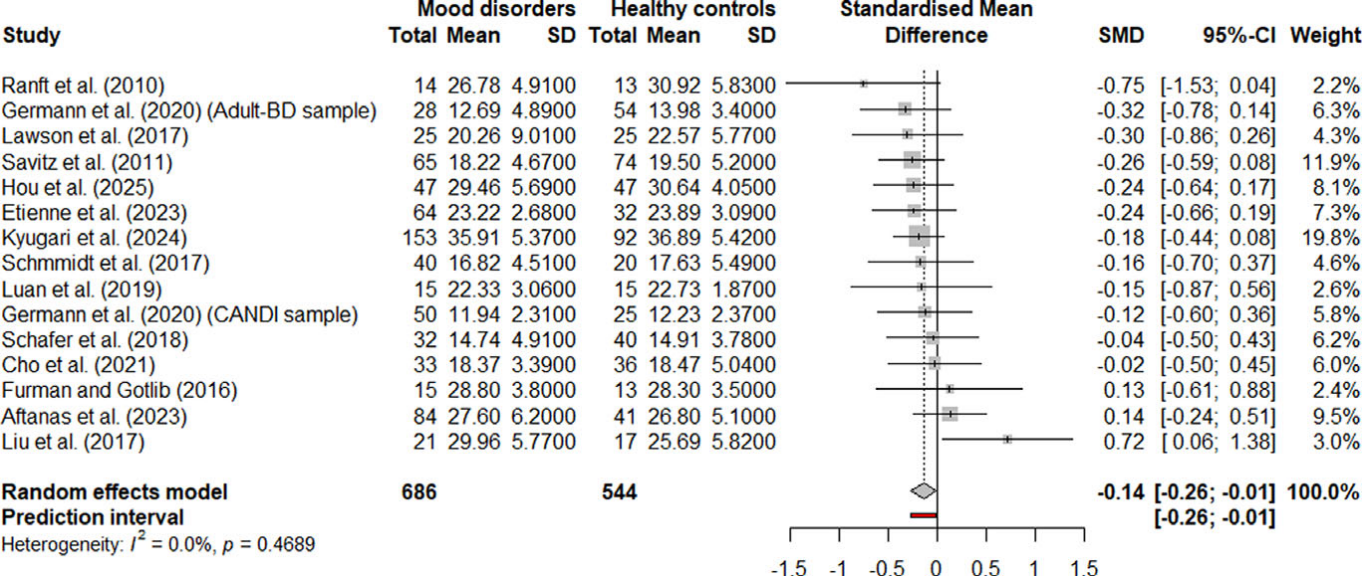
Forest plot for the meta-analysis comparing the volume of the left habenula in patients with a mood disorder versus healthy controls (HCs).

For the left habenula volume, the global meta-analysis included 15 samples (1,230 participants: 686 with a mood disorder, 544 HCs) (Figure 2). A random-effects model showed a small but significant volume reduction in mood disorders (*g* = −0.1367, 95% CI [−0.2618, −0.0116], *p* = .0344). The effect size ranged from −0.1654 to −0.1205 in the leave-one-out analysis (Supplementary Figure S19). In six (Etienne et al., 2023; Germann et al., 2020; Hou et al., 2025; Kyuragi et al., 2024; Lawson et al., 2017; Savitz et al., 2011) out of the 15 samples, removal of the individual sample rendered the meta-analytic effect non-significant, indicating limited robustness of the observed effect. Moreover, after applying a 5% FDR correction, the adjusted p-value was 0.0992, rendering the effect non-significant. Between-study heterogeneity was low (*τ* ^2^ < 0.0001, 95% CI [0.0000, 0.1311]), with an *I* ^2^ value of 0.0% (95% CI [0.0%, 53.6%]), indicating low heterogeneity and suggesting consistency across studies. The Q-test did not indicate significant heterogeneity (*Q* = 13.74, *df* = 14, *p* = .4689). The 95% prediction intervals ranged from −0.2630 to −0.0104, suggesting that future studies are also likely to observe a negative effect. Neither Egger’s test nor the funnel plot (Supplementary Figure S25) showed evidence of publication bias (*t* = 0.51, *p* = 0.6176), and trim-and-fill analysis suggested no missing studies.

**Figure 3.**
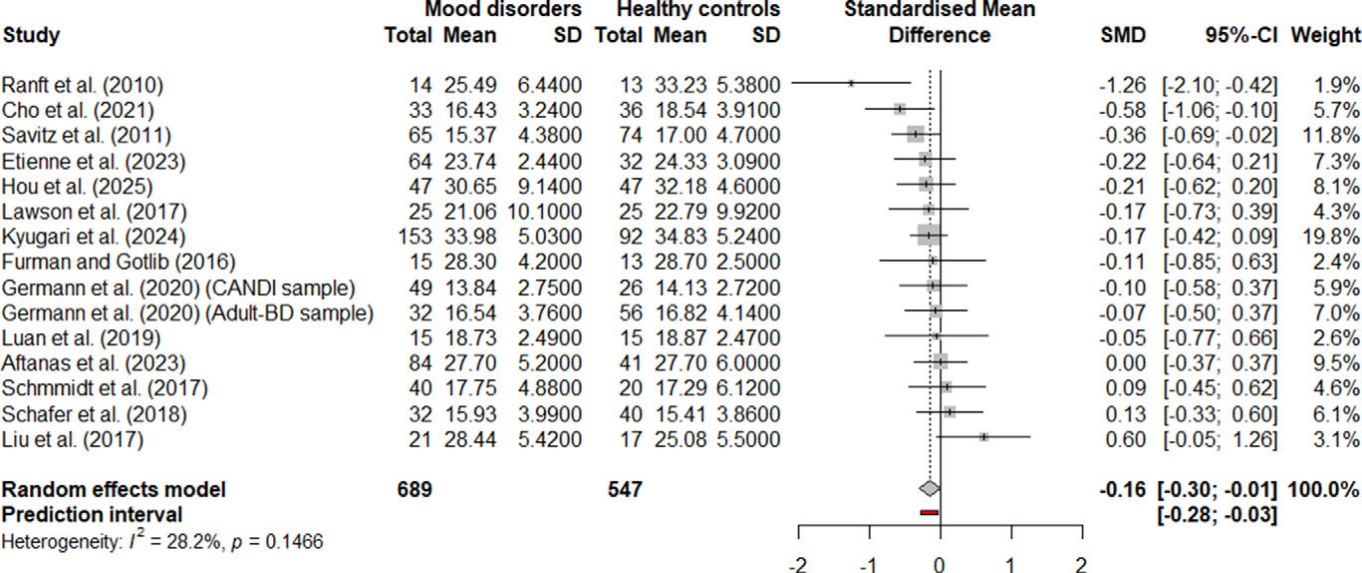
Forest plot for the meta-analysis comparing the volume of the right habenula in patients with a mood disorder versus healthy controls (HCs).

For the right habenula volume, the global meta-analysis included 15 samples (1,236 participants: 689 with a mood disorder, 547 HCs) (Figure 3). A random-effects model showed a small but significant volume reduction in mood disorders (*g* = −0.1562, 95% CI [−0.3049, −0.0074], *p* = .0409). The effect size ranged from −0.1804 to −0.1296 in the leave-one-out analysis (Supplementary Figure S20). In seven (Cho et al., 2021; Etienne et al., 2023; Germann et al., 2020; Hou et al., 2025; Kyuragi et al., 2024; Lawson et al., 2017; Savitz et al., 2011) out of the 15 samples, removing that individual sample rendered the meta-analytic effect non-significant, indicating limited robustness of the observed effect. After FDR correction, the adjusted *p*-value was 0.0992, rendering the result non-significant. Between-study heterogeneity was low (*τ* ^2^ < 0.0001, 95% CI [0.0000, 0.2584]), with an *I* ^2^ value of 28.2% (95% CI [0.0%, 61.4%]), indicating low to moderate heterogeneity. The Q-test did not indicate significant heterogeneity (*Q* = 19.50, *df* = 14, *p* = .1466). The 95% prediction interval ranged from −0.2823 to −0.0301, suggesting that future studies are also likely to observe a negative effect. Neither Egger’s test nor the funnel plot (Supplementary Figure S26) showed evidence of publication bias (*t* = 0.03, *p* = 0.9735). Trim-and-fill analysis indicated three potentially missing studies. After imputing these studies, the adjusted meta-analytic effect size remained statistically significant and even shifted slightly toward a more negative value (*g* = −0.2247, 95% CI [−0.3958, −0.0535], *p* = 0.0131). However, after FDR correction, the *p*-value rose to 0.0786, rendering the result non-significant.

Separate meta-analyses for MDD and BD showed no significant effects for either habenula (Supplementary Figures S3–S6).

### Secondary analyses

We conducted separate meta-analyses to examine left and right habenula volumes in individuals with a mood disorder (i.e. MDD or BD), MDD, and BD, stratified by segmentation method (automatic versus manual). Additionally, we carried out six tests of subgroup differences based on the segmentation method for each diagnostic group (mood disorder, MDD, and BD) and hemisphere (left and right) (Supplementary Figures S7–S12). None of the separate meta-analyses or tests of subgroup differences remained statistically significant after FDR correction. We then repeated the same set of analyses for medication status (medicated versus unmedicated) (Supplementary Figures S13–S18), and observed a similar pattern, with no statistically significant results following correction. Meta-regressions tested MRI resolution as a moderator; it was not significant for either habenula in mood disorders (left: *β* = −0.1201, *p* = .3317; right: *β* = 0.0571, *p* = .6556) or in MDD-only analyses (left: *β* = −0.4551, *p* = .2600; right: *β* = −0.0443, *p* = .9179).

## Discussion

Our meta-analyses offer the first comprehensive synthesis of evidence on habenula volume in mood disorders. In our global meta-analyses – pooling data from MDD and BD – we found small reductions in both left and right habenula volume, though these did not survive FDR correction. Separate analyses of MDD and BD showed no significant group differences, but all four subgroup meta-analyses revealed nonsignificant volume reductions, with effect sizes ranging from −0.09 (left) to −0.15 (right) in MDD, and − 0.13 (left) to −0.23 (right) in BD. Although the results from our six meta-analyses do not provide conclusive evidence for reduced habenula volume in mood disorders, they consistently point toward a possible small reduction, as all analyses showed effects in the same direction – albeit non-significant – and the two global meta-analyses reached statistical significance prior to FDR correction.

To assess publication bias, we used Egger’s test, funnel plots, and the trim-and-fill method. Egger’s test showed no evidence of bias in the global meta-analyses. The trim-and-fill procedure identified no missing studies for left habenula volume but suggested possible bias for the right. After imputing studies, the adjusted effect size for right habenula volume remained statistically significant and shifted slightly toward a more negative value (*g* = −0.2247), further supporting the possibility of a small volumetric reduction in mood disorders. Overall, our findings raise no major concerns about publication bias and may even suggest a bias toward reporting increased volume in mood disorders.

As registered, we conducted subgroup analyses by segmentation method and medication status, as well as meta-regressions with MRI resolution as a moderator. No comparisons between automatic and manual segmentation were significant, nor were any meta-analyses based on subgroups after FDR correction. MRI resolution was not a significant moderator. Overall, results from the subgroup analyses and meta-regressions should be interpreted cautiously due to limited power – no analysis included more than 14 samples.

Although our findings did not survive FDR correction, reduced habenula volume in mood disorders would align with emerging evidence from animal models. Two studies have examined habenula volume in depression models: Yang et al. (2019) reported reduced total volume in a lipopolysaccharide model of depression, while Jacinto et al. (2017) found bilateral volume reductions following chronic stress, along with a decreased number of neurons in the right MHb and a decreased number of glial cells bilaterally in the LHb. Human studies also suggest that effective treatment may increase habenula volume. Etienne et al. (2024) observed significant volume increases after 3 months of venlafaxine in MDD patients, and Elias et al. (2022) reported bilateral volume increases in responders to subcallosal cingulate deep brain stimulation, with clinical response linked to volume change. Sartorius et al. (2016) found increased gray matter volume in both habenulae after electroconvulsive therapy in treatment-resistant depression, though interpretation is limited by the lack of correlation with clinical scores. In contrast, Anand et al. (2020) reported reduced habenula volume after lithium treatment in BD. Overall, our findings align with several lines of evidence – both in humans and animals – suggesting reduced habenula volume in mood disorders.

The neurotrophic hypothesis of depression may offer a framework for understanding the putative link between habenula volume and mood disorders (Duman & Monteggia, 2006). It posits that stress – and the resulting elevation of glucocorticoids – reduces expression of neurotrophic factors, particularly BDNF, in key limbic regions (Duman & Monteggia, 2006). The resulting BDNF deficiency is thought to drive structural atrophy and neuronal and glial loss, particularly in the hippocampus and mPFC (Duman, Aghajanian, Sanacora, & Krystal, 2016; Duman & Monteggia, 2006; Pittenger & Duman, 2008). Similar mechanisms could plausibly affect the habenula as well, with elevated glucocorticoids and reduced BDNF signaling contributing to its structural alterations.

In both animal models and human studies, increased glucocorticoid exposure is associated with hippocampal (Lupien et al., 1998; Zhang, Zhao, & Wang, 2015) and mPFC (Kim et al., 2014; Stomby et al., 2016) atrophy. Supporting a similar pattern in the habenula, Jacinto et al. (2017) found strong negative correlations between systemic glucocorticoid levels and bilateral habenula volume. However, it remains unclear whether this reflects a direct effect, as glucocorticoid receptors have not been identified in the habenula (Aronsson et al., 1988). Another possible contributor to stress-related habenula atrophy is corticotropin-releasing hormone (CRH), a key regulator of the stress response (Sukhareva, 2021), with known receptor expression in the habenula (Authement et al., 2018; Justice, Yuan, Sawchenko, & Vale, 2008). In the hippocampus, CRH signaling via CRH receptor 1 (CRHR1) has been implicated in stress-induced atrophy (Maras & Baram, 2012), with evidence suggesting that hippocampal neurons themselves are a primary source of CRH acting locally on CRHR1 receptors, contributing to structural degradation (Chen, Andres, Frotscher, & Baram, 2012). A similar mechanism may operate in the habenula. The rodent LHb has been shown to be highly responsive to CRH (Authement et al., 2018), and CRHR1 expression has been identified in this region (Justice et al., 2008). Strikingly, retrograde tracing in CRH-Cre mice has shown that the LHb itself is a major source of CRH projections to the LHb (Flerlage et al., 2025), as is the case for the hippocampus. These parallels raise the possibility that locally driven CRH–CRHR1 signaling within the habenula may also contribute to stress-related atrophy.

Further supporting the notion that the neurotrophic hypothesis of depression may also apply to the habenula, several studies show that stress and antidepressant treatments modulate BDNF expression in this region. Zhang et al. (2022) reported significantly reduced BDNF levels in the habenula of rats exposed to chronic restraint stress, with ketamine treatment increasing habenula BDNF levels. Similarly, Tong et al. (2023) found decreased LHb BDNF expression in rats subjected to chronic unpredictable mild stress, reversed by acupuncture or fluoxetine treatment. Consistent with this, Sachs et al. (2015) observed elevated levels of BDNF mRNA in the habenula of mice after fluoxetine administration. In contrast, however, Lei et al. (2020) reported that the antidepressant effects of Rislenemdaz may involve downregulation of BDNF in the LHb, suggesting that the relationship between BDNF and antidepressant mechanisms in the habenula may be complex and context-dependent.

Lastly, inflammation represents another potential mechanism hypothesized to contribute to structural alterations in the hippocampus and mPFC (Belleau et al., 2019), and may similarly impact the habenula. Chronic unpredictable stress has been shown to increase the production of pro-inflammatory cytokines in both the hippocampus (Iwata et al., 2016) and the mPFC (Pan, Chen, Zhang, & Kong, 2014). Notably, similar stress paradigms also upregulate inflammatory cytokine expression in the habenula (Wang et al., 2022), pointing to inflammation as a common downstream consequence of stress exposure that may contribute to structural changes across multiple mood-related brain regions.

While our discussion highlights several neurobiological mechanisms that could plausibly underlie structural changes in the habenula, we must emphasize that our findings did not survive correction for multiple comparisons. These explanations should therefore be considered preliminary and hypothesis-generating, as future studies are needed to confirm whether habenula volume reductions are reliably observed in mood disorders.

### Limitations and future directions

One limitation of our study is that research on habenula volume in mood disorders is still relatively nascent, resulting in a limited number of eligible studies. Notably, the effect sizes observed in our global analyses – *g* = −0.14 for the left habenula and −0.16 for the right – were similar in magnitude to those reported for hippocampal volume in a large meta-analysis of 1,728 MDD patients and 7,199 HCs (*d* = −0.14) (Schmaal et al., 2016). However, our small number of samples likely limited statistical power, preventing effects from surviving FDR correction. As more data accumulate, future meta-analyses may be positioned to detect reliable effects. Additionally, our subgroup analyses and meta-regression were underpowered, limiting our ability to draw conclusions about potential moderators of effect size. While we attempted to examine the impact of medication status, our analysis was limited both by the small number of available samples and by the fact that most studies did not report data stratified by medication status. As a result, we were only able to compare unmedicated samples to those that included at least some medicated participants. Existing evidence suggests that habenula volume may increase following antidepressant treatment, indicating that medication exposure may have influenced our results (Elias et al., 2022; Etienne et al., 2024; Sartorius et al., 2016). To enhance the ability of future meta-analyses to detect medication-related effects, it is crucial that primary studies systematically report findings separately for medicated and unmedicated participants. Another important factor likely affecting the magnitude of volume reductions is illness severity. Indeed, volume reductions in the hippocampus and mPFC have been consistently linked to markers of illness progression, including recurrent episodes, longer illness duration, and treatment resistance (Schmaal et al., 2016). Unfortunately, very few studies in our dataset reported sufficient detail on illness severity, precluding us from examining its role in habenula volume. Future research should prioritize the collection of such clinical variables to better understand their impact.

Finally, a broader methodological limitation in the field is the difficulty of accurately segmenting the habenula due to its small size and poor contrast with adjacent structures (Strotmann et al., 2013), which may limit measurement precision. To address this, future studies should prioritize the use of ultra-high-field MRI (e.g. 7 T) to improve spatial resolution and contrast (Calabro et al., 2024; Sclocco, Beissner, Bianciardi, Polimeni, & Napadow, 2018). Only two included samples employed 7 T imaging, underscoring the need for wider adoption of this technology. Another limitation of the field is the reliance on manual habenula segmentation, which may be prone to imprecision due to the small size of the structure and the requirement for detailed anatomical expertise. A promising avenue for future research is the application of automated habenula segmentation methods (e.g. Kim & Xu, 2022; Kyuragi et al., 2024) to large databases of patients with a mood disorder, as this approach would enable the analysis of larger samples by overcoming the time constraints of manual segmentation, thereby increasing statistical power.

## Conclusion

In conclusion, although small reductions in left and right habenula volumes were observed in mood disorders, these did not survive FDR correction. Furthermore, separate analyses for BD and MDD showed no significant group differences in habenula volumes for either hemisphere. Interestingly, the effect sizes observed were similar in magnitude to those reported for hippocampal volume reductions in depression, suggesting that our meta-analyses may have lacked sufficient power to detect statistically significant effects. Nonetheless, the consistent direction of effects across analyses hints at a true diminution in habenula volume in mood disorders. Future studies with ultra-high-field MRI, automated segmentation, and larger samples are needed.

## Supporting information

10.1017/S0033291726103730.sm001 Fortin et al. supplementary material Fortin et al. supplementary material

## Data Availability

Raw data and analysis scripts are available at: https://osf.io/fc95y/overview.

## Supplementary material

The supplementary material for this article can be found at http://doi.org/10.1017/S0033291726103730.
