# Investigation of habenula volume in mood disorders: A meta-analytic study – CORRIGENDUM

**DOI:** 10.1017/S0033291726104656

**Published:** 2026-07-31

**Authors:** Jean-Simon Fortin, Charles Parisien, Catherine Parent, Sébastien Hétu

This article was published with an incorrectly ordered list of authors. The correct author order is: Jean-Simon Fortin, Charles Parisien, Catherine Parent, Sébastien Hétu. The authors apologise for the mistake.
