# Supplementary material for: Investigation of habenula volume in mood disorders: A meta-analytic study
Source: Psychol Med. 2026 Mar 30;56:e82. doi: 10.1017/S0033291726103730 (PMC13079212; doi:10.1017/S0033291726103730)
Supplement: Fortin et al. supplementary material [file S0033291726103730sup001.docx]

**Deviations from registered protocol**

We made several deviations from the registered protocol. First, we performed a trim-and-fill analysis to assess potential publication bias, which was not specified in the original plan. Second, we extracted information on scanner magnet strength (e.g., 1.5T vs. 3T) for descriptive purposes, although we had initially omitted this from the registered protocol. Finally, we performed and reported subgroup analyses even when the number of included studies was fewer than 10 (*k* < 10), despite our initial intention to do so only when *k* exceeded 10. We made this decision because our registered protocol also stated that we would conduct separate meta-analyses when the number of samples in a subgroup exceeded 2 (*k* > 2). Given the small number of samples per subgroup in our analyses, we opted to conduct these analyses within a subgroup framework that assumes a common estimate of between-study heterogeneity (*τ²*) across subgroups. When subgroup-specific estimates of *τ²* are likely to be unstable (e.g., when *k* *≤* 5), pooling *τ²* across subgroups is thought to provide more reliable estimates (1). Because we assumed a common *τ²*, the *dmetar* R package automatically generated statistical tests of subgroup differences in the output plots. Since these comparisons were already computed and visualized, we chose to report them—while clearly acknowledging the limited interpretability of subgroup analyses with low power.

**Supplemental Methods and Materials**

**Literature Search**

For Google Scholar, only the first 200 results were screened, consistent with the recommendations of Bramer et al. (2) for biomedical systematic reviews. Database selection was also guided by these recommendations. In addition to the database search, we also screened the reference lists of all included articles to identify any additional eligible studies, although none was found.

Furthermore, to retrieve additional data, we undertook three strategies. First, we contacted leading researchers in the field of human habenula and mood disorders to inquire about any unpublished data relevant to our meta-analysis. Second, we reached out to either the senior or corresponding author (whichever was deemed more appropriate) of all studies included in our meta-analysis to request relevant unpublished data. Third, we contacted authors of studies that appeared to have collected habenula volume data for both HCs and individuals with mood disorders, but for which essential data to calculate effect sizes were not reported in the publication. To contact authors, we followed a standardized procedure. An initial email was sent outlining our data request. If no response was received within approximately two weeks, a reminder was sent. After an additional two weeks without a reply (i.e., four weeks in total), the request was considered closed, and any data received after this point were not included in the meta-analysis. In total, we sent three emails to authors of studies that appeared to have collected relevant data but did not report sufficient information to calculate effect sizes. We received two responses and obtained usable data from one author. We also contacted 12 authors of studies already included in the meta-analysis to request any unpublished habenula volume data; five authors responded, and none provided data. Finally, we contacted three leading researchers in the field for relevant unpublished datasets, receiving one response and no. In one instance, we also contacted a senior author to clarify whether two publications (3, 4) included overlapping samples; no response was received.

Below are the full search strategies for all databases:

***Ovid APA PsycInfo <no limit to April 8th 2025>***

1. habenula*.mp.

2. size.mp.

3. volum*.mp.

4. mri.mp.

5. "Magnetic resonance imaging".mp.

6. 2 or 3 or 4 or 5

7. exp affective disorders/

8. depress*.mp.

9. "major depressive disorder*".mp.

10. "mood disorder*".mp.

11. suicid*.mp.

12. "bipolar disorder*".mp.

13. bipolar*.mp.

14. exp Bipolar Disorder/

15. Anhedonia/ or anhedoni*.mp.

16. 7 or 8 or 9 or 10 or 11 or 12 or 13 or 14 or 15

17. 1 and 6 and 16

***Ovid Embase <no limit to April 8th 2025>***

1. habenula*.mp.

2. size.mp.

3. volum*.mp.

4. mri.mp.

5. "Magnetic resonance imaging".mp.

6. 2 or 3 or 4 or 5

7. exp mood disorder/

8. depress*.mp.

9. "major depressive disorder*".mp.

10."mood disorder*".mp.

11. suicid*.mp.

12. "bipolar disorder*".mp.

13. bipolar*.mp.

14. exp bipolar disorder/

15. anhedoni*.mp.

16. anhedonia/

17. 7 or 8 or 9 or 10 or 11 or 12 or 13 or 14 or 15 or 16
18. 1 and 6 and 17

***Ovid MEDLINE(R) ALL <no limit to April 8th 2025>***

1. habenula*.mp.

2. size.mp.

3. volum*.mp.

4. mri.mp.

5. "Magnetic resonance imaging".mp.

6. 2 or 3 or 4 or 5

7. exp Mood Disorders/

8. depress*.mp.

9. "major depressive disorder*".mp.

10. "mood disorder*".mp.

11. suicid*.mp.

12. "bipolar disorder*".mp.

13. bipolar*.mp.

14. exp Bipolar Disorder/

15. anhedoni*.mp.

16. Anhedonia/

17. 7 or 8 or 9 or 10 or 11 or 12 or 13 or 14 or 15 or 16

18. 1 and 6 and 17

***Web of Science Core Collection (Clarivate)***

TS=(habenula*) AND TS=(size OR volum* OR mri OR "magnetic resonance imaging") AND TS=(depress* OR "major depressive disorder*" OR "mood disorder*" OR suicid* OR "bipolar disorder*" OR bipolar* OR anhedoni*)

***Google Scholar***

habenula AND (size OR volum* OR MRI OR "magnetic resonance imaging") AND (depression OR "major depressive disorder*" OR "mood disorder*" OR suicid* OR "bipolar disorder" OR bipolar* OR anhedoni*) 200 first results.

**Study Selection**

During full-text screening, two publications—Cho et al. (4) and Lim et al. (3)—were identified as likely reporting overlapping samples. Although we did not receive confirmation from the corresponding author, the demographic characteristics were highly similar and the studies originated from the same research group, leading us to conclude that the samples overlapped. The study by Cho et al. (4) was retained because it directly investigated habenula volume differences in individuals with depression, while Lim et al. (3) focused on the methodological development of a habenula segmentation approach. Consequently, Lim et al. (3) was manually tagged and removed as a duplicate.

Inter-rater reliability for both the title and abstract screening stage and the full-text screening stage was assessed using Cohen’s Kappa. During the title and abstract screening stage, 383 records were screened, with an observed proportionate agreement of 95.6% and a Cohen’s Kappa of 0.62, indicating substantial agreement between raters (5). During the full-text screening stage, 32 articles were assessed, yielding a proportionate agreement of 84.4% and a Cohen’s Kappa of 0.69, also indicating substantial agreement.

**Data Extraction**

Key characteristics were systematically extracted from each included dataset. The following details were recorded: first author, publication year, clinical diagnosis of the study groups (e.g., MDD or BD), sample size, sex distribution, average age, medication status, and the country where data collection took place (Table 1). When applicable, demographic information such as sample size, sex, and mean age was charted separately for clinical and control groups. In addition, the following details were extracted for each dataset: method used to obtain the volume of the habenula (e.g., structural MRI versus post-mortem method), MRI image resolution, scanner magnetic field strength (e.g., 3T versus 7T), and method used to segment the habenula (e.g., manual or automatic). When data were only available in graphical form, we used WebPlotDigitizer (6) to extract the values. This applied to three studies (7-9), covering a total of four samples. In the paper by Germann et al. (7), we were not able to retrieve all data. The number of participants retrieved were different between the left and right habenula. Therefore, the number of participants included in our analyses will differ slightly for left and right habenula where the samples by Germann et al. (7) are included in analyses.

In some cases, we needed to combine reported data across subgroups to allow for consistent pairwise comparisons. For example, when studies reported separate data for multiple clinical subgroups (e.g., medicated and unmedicated patients), these groups were combined into a single clinical group to compare against the HC group. One study also reported habenula volumes separately for the medial and lateral subdivisions (10). In this case, we pooled the data to obtain the total habenula volume.

When studies reported data separately for medicated and unmedicated patients, we treated these as independent samples in our subgroup analyses based on medication status. In such cases, the number of HCs was divided equally between the two patient groups to avoid double-counting.

We now detail the calculations applied to each study where data transformation was necessary.

***Savitz et al. (11):***

For meta-analyses combining both MDD and BD, we pooled three groups: unmedicated BD, medicated BD, and MDD. A fourth group—patients with remitted depression—was excluded. Because more than two groups were combined, we applied Formula 1 sequentially to compute the pooled means and standard deviations: first combining the unmedicated and medicated BD groups to create a composite BD group, then combining this with the MDD group. For the meta-analyses limited to BD, we combined the unmedicated and medicated BD groups using the same formula.

In the subgroup analyses based on medication status, for the meta-analyses comparing mood disorder patients to HCs, we pooled the MDD and unmedicated BD groups—since both were unmedicated. Formula 1 was used here as well. Additionally, because this study contributed two samples to the medication status analyses (one medicated and one unmedicated), the number of HCs was divided equally between the medicated and unmedicated groups to avoid double-counting. Similarly, for the BD versus HCs meta-analyses, in the subgroup analyses based on medication status, this study contributed two samples. Thus, the number of HCs was also divided equally between the medicated and unmedicated groups.

***Schmidt et al. (12)***

In this study, data were reported separately for medicated and unmedicated MDD groups. To include this study in the meta-analyses pooling both MDD and BD, as well as in the MDD-only analyses, we combined the two MDD groups using Formula 1. For subgroup analyses based on medication status, this study contributed two samples (medicated and unmedicated). To avoid double-counting, the number of HCs was divided equally between the two patient groups.

***Ranft et al. (10)***

This study reported habenula volumes separately for the medial and lateral subdivisions. To calculate the means for the total habenula volume, we again used Formula 1. To calculate the pooled standard deviations for the total habenula volume, we applied Formula 2. Formula 2 requires an assumption about the correlation between the medial and lateral habenula volumes. We assumed a correlation coefficient of 0.5.


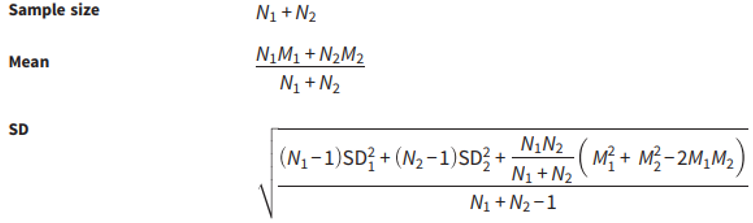


**Formula 1.** Formula taken from Table 6.5a of the *Cochrane Handbook for Systematic Reviews of Interventions* (13).


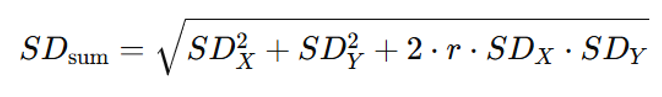


**Formula 2.** This formula can be easily derived from box 29.1 in the Borenstein et al. (14) handbook.

**Quality Assessment**

The NIH Quality Assessment Tool for Observational Cohort and Cross-Sectional Studies is specifically designed to evaluate the internal validity of studies included in systematic reviews. The tool comprises 14 questions allowing to assess the quality of the studies. For each of these questions, one option was chosen between yes, no, not applicable (NA), or not reported (NR). Subsequently, for each study, the methodological quality was subjectively evaluated and categorized as either good, fair, or poor.

**Data Analysis**

Meta-analyses were conducted using the dmetar R package (15). The heterogeneity variance *τ²* was estimated using the restricted maximum likelihood (REML) method (16). To construct the confidence interval around the pooled effect, we applied the Knapp-Hartung adjustment (17). Heterogeneity was assessed using Cochran’s Q test, the *I²* statistic, and 95% prediction intervals. We interpreted heterogeneity following common benchmarks for the *I²* statistic: 25% indicating low heterogeneity, 50% moderate heterogeneity, and 75% substantial heterogeneity (18). As a sensitivity analysis, we performed a leave-one-out analysis by recalculating the meta-analyses multiple times, each time excluding one sample. This allowed us to assess how the removal of each individual sample influenced the overall results. We also conducted sensitivity analyses focusing on the standard deviations that were estimated in Ranft et al. (10).

We assessed publication bias by testing for funnel plot asymmetry using Egger’s test. We present funnel plots when *k* > 10. Given that Egger’s test has low statistical power when the number of samples is small, we used a significance threshold of p < 0.10, as recommended by Egger et al. (19). Additionally, we only conducted this test when the number of samples was at least 10 (*k* ≥ 10), in line with recommendations by Sterne et al. (20). Although not initially specified in our registered analysis plan, we also applied the trim-and-fill method. This approach estimates the number of potentially missing studies due to publication bias and imputes them to produce a bias-adjusted estimate of the overall effect size by rebalancing the funnel plot.

**Supplemental Results**

**Study Characteristics**

The included studies were published between 2010 and 2024, with one preprint released in 2025 (Figure S1). The countries contributing the greatest number of samples were the United States (n = 4) and China (n = 3) (Figure S2). For the global meta-analyses, control group sample sizes ranged from 13 to 92 participants, with a mean of 36.27 ± 23.05 for the left habenula and 36.47 ± 23.13 for the right habenula. In mood disorder groups, sample sizes ranged from 14 to 153 participants, with a mean of 45.73 ± 36.18 for the left habenula and 45.93 ± 36.05 for the right. For the global meta-analyses, among studies reporting sex data, 57.5% of HCs (207 out of 360) and 61.13% of mood disorder participants (269 out of 440) were female.


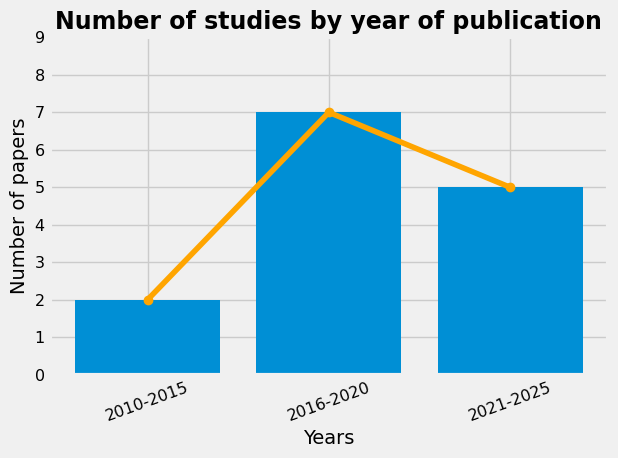


**Figure S1.**


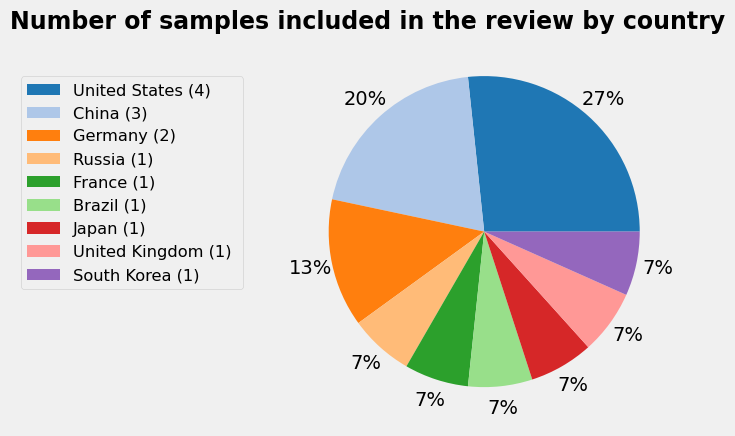


**Figure S2.**

**Meta-Analyses for MDD**

A total of 11 independent samples comprising 937 participants (525 with MDD, 412 HCs) were included in the meta-analysis of left habenula volume. Results from a random-effects model revealed no statistically significant difference in right habenula volume among individuals with MDD compared to HCs (*g* = -0.0879, 95% CI [-0.2372, 0.0614], *p* = 0.2188, *p_FDR_*=0.2625) (Figure S3). The effect size ranged from -0.1223 to -0.0542 in the leave-one-out analysis (Figure S21), and it never reached significance regardless of which sample was left out. The between-study heterogeneity variance was estimated at *τ²* < 0.0001 (95% CI [0.0000, 0.1605]), with an *I²* value of 0.0% (95% CI [0.0%, 60.2%]), indicating low heterogeneity and suggesting consistency across studies. The Q-test did not indicate significant heterogeneity (*Q* = 9.68, *df* = 10, *p* = .4689). The 95% prediction interval ranged from –0.2397 to 0.0639. Egger’s test for funnel plot asymmetry did not indicate evidence of publication bias (*t* = 1.18, *p* = 0.2680). The trim-and-fill method did not suggest any missing studies due to funnel plot asymmetry for the left habenula volume analysis. The adjusted meta-analytic effect size remained unchanged, with no added studies imputed.

A total of 11 independent samples comprising 937 participants (525 with MDD, 412 HCs) were included in the meta-analysis of right habenula volume. Results from a random-effects model revealed no statistically significant difference in right habenula volume among individuals with MDD compared to HCs (*g* = -0.1453, 95% CI [-0.2984, 0.0078], *p* = 0.0605, *p_FDR_* = 0.0992) (Figure S4). The effect size ranged from -0.1777 to -0.1093 in the leave-one-out analysis (Figure S22). Across the leave-one-out analysis, statistical significance (uncorrected p < .05) was observed only when the study by Liu et al. (9) was excluded. This suggests that the overall non-significant effect is generally stable and not driven by any single study, with the exception of this case. The between-study heterogeneity variance was estimated at *τ²* < 0.0001 (95% CI [0.0000, 0.1714]), with an *I²* value of 1.5% (95% CI [0.0%, 60.8%]), indicating low heterogeneity and suggesting consistency across studies. The Q-test did not indicate significant heterogeneity (*Q* = 10.16, *df* = 10, *p* = .4270). The 95% prediction interval ranged from –0.2972 to 0.0066. Egger’s test for funnel plot asymmetry did not indicate evidence of publication bias (*t* = 0.84, *p* = 0.4253). The trim-and-fill method suggested the presence of three potentially missing studies due to funnel plot asymmetry for the right habenula volume analysis. After imputing these studies, the adjusted meta-analytic effect size became statistically significant (*g* = -0.2324, 95% CI [-0.4123, -0.0524], *p* = 0.0153). However, this result did not remain significant after FDR correction (adjusted *p_FDR_* = 0.0818).

***
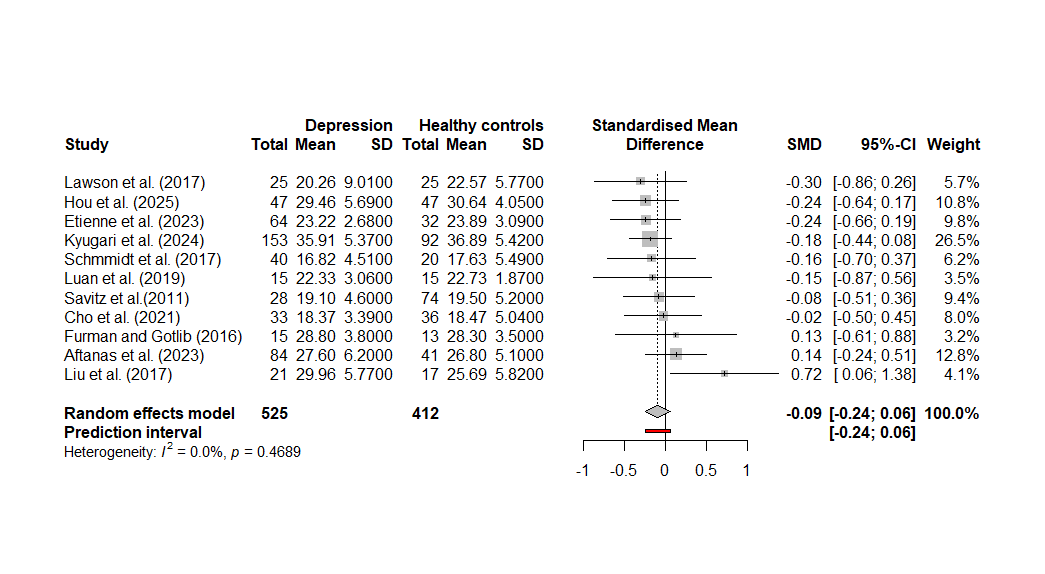
***

**Figure S3.** Forest plot of the meta-analysis comparing the volume of the left habenula in patients with MDD versus HCs.

***
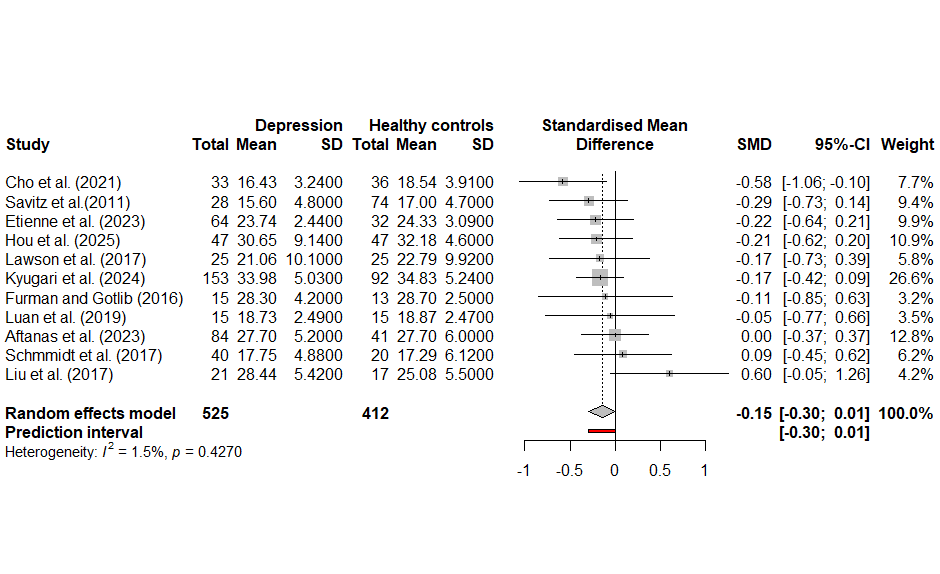
***

**Figure S4.** Forest plot of the meta-analysis comparing the volume of the right habenula in patients with MDD versus HCs.

**Meta-Analyses for BD**

A total of four independent samples comprising 340 participants (147 with BD, 193 HCs) were included in the meta-analysis of left habenula volume. Results from a random-effects model revealed no statistically significant difference in left habenula volume among individuals with BD compared to HCs (*g* = -0.2327, 95% CI [-0.4943, 0.0289], p= 0.0661, *p_FDR_*=0.0992) (Figure S5). The effect size ranged from -0.2910 to -0.1636 in the leave-one-out analysis (Figure S23), and it never reached significance regardless of which sample was left out. The between-study heterogeneity variance was estimated at *τ²* < 0.0001 (95% CI [0.0000, 0.3150]), with an *I²* value of 0.0% (95% CI [0.0%, 84.7%]), indicating low heterogeneity and suggesting consistency across studies. The Q-test did not indicate significant heterogeneity (*Q* = 1.56, *df* = 3, *p* = .6688). The 95% prediction interval ranged from –0.5957 to 0.1302. Because there was only 4 samples, we did not conduct a publication bias analysis.

A total of four independent samples comprising 346 participants (150 with BD, 196 HCs) were included in the meta-analysis of right habenula volume. Results from a random-effects model revealed no statistically significant difference in right habenula volume among individuals with BD compared to HCs (*g* = -0.1306, 95% CI [-0.4896, 0.2285], *p* = 0.3309, *p_FDR_*= 0.3309) (Figure S6). The effect size ranged from -0.2086 to -0.0156 in the leave-one-out analysis (Figure S24), and it never reached significance regardless of which sample was left out. The between-study heterogeneity variance was estimated at *τ²* = 0.0045 (95% CI [0.0000, 0.6158]), with an *I²* value of 1.4% (95% CI [0.0%, 84.9%]), indicating low heterogeneity and suggesting consistency across studies. The Q-test did not indicate significant heterogeneity (*Q* = 3.04, *df* = 3, *p* = .3851). The 95% prediction interval ranged from –0.5610 to 0.2999. Because there were only 4 samples, we did not conduct a publication bias analysis.


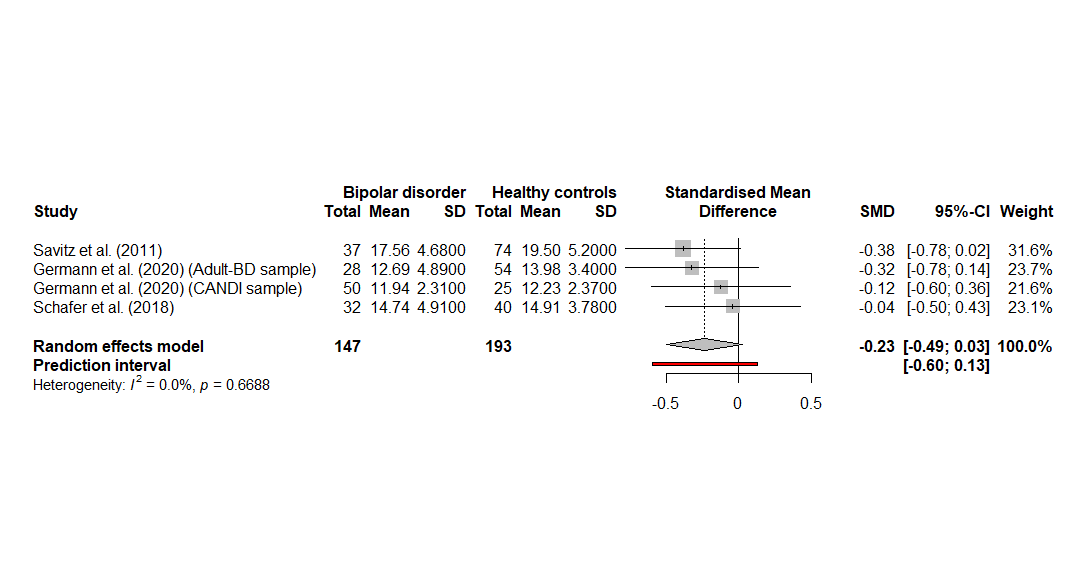
**Figure S5.** Forest plot of the meta-analysis comparing the volume of the left habenula in patients with BD versus HCs.

**
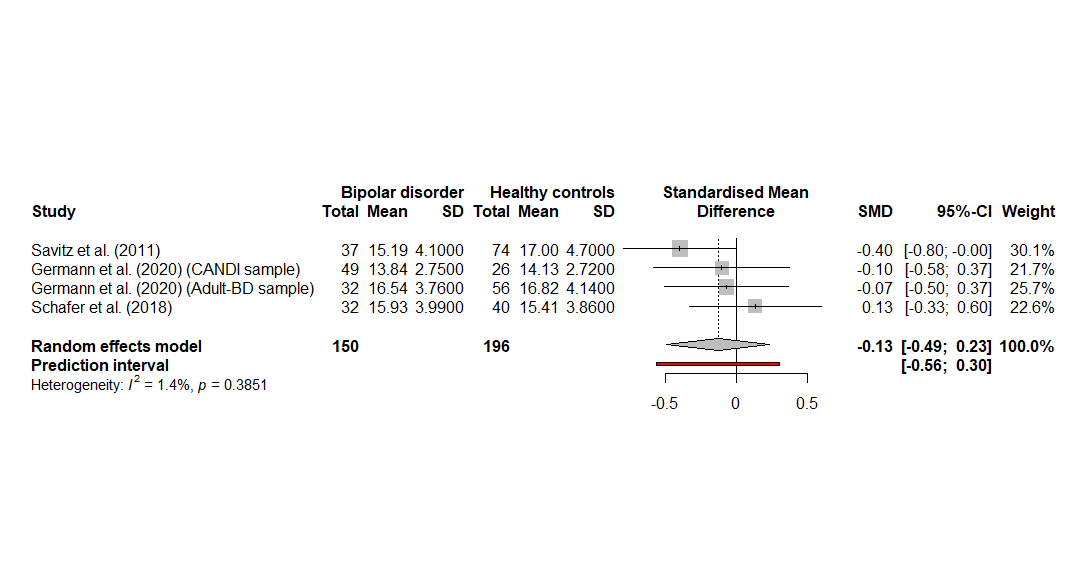
**

**Figure S6.** Forest plot of the meta-analysis comparing the volume of the right habenula in patients with BD versus HCs.

**Subgroup Analyses Based on Segmentation Method**


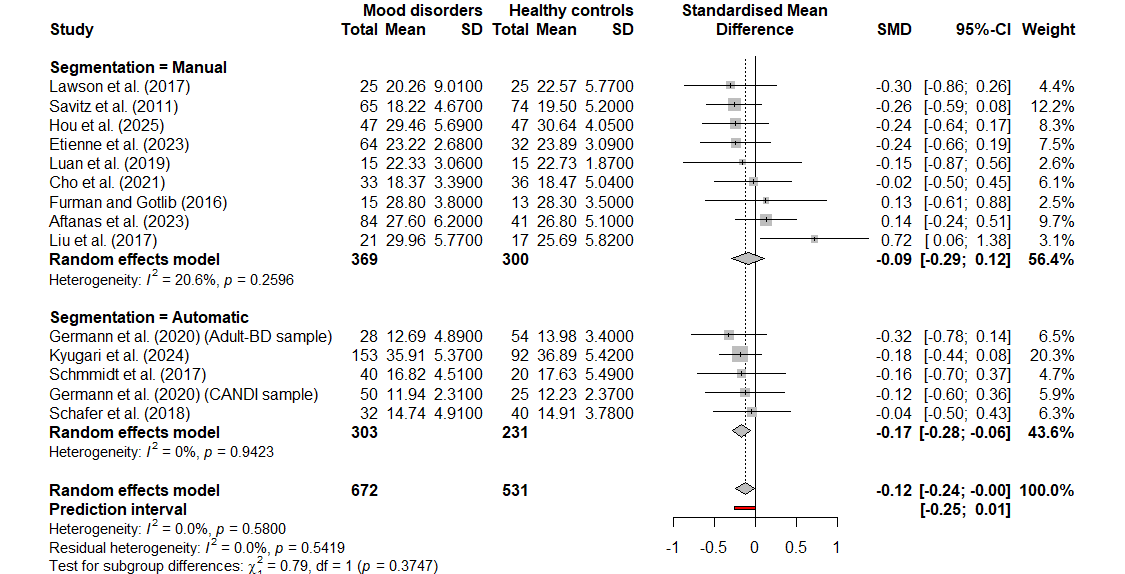


**Figure S7.** Forest plot presenting the subgroup analysis based on segmentation method for left habenula (individuals with a mood disorder versus HCs). Among samples using manual segmentation, there was no significant difference in habenula volume (*g* = -0.0855, 95% CI [-0.2907, 0.1197], *p* = 0.3647). Among samples using automatic segmentation, the left habenula was significantly smaller in patients with a mood disorder (*g* = -0.1720, 95% CI [-0.2818, -0.0621], *p* = 0.0122). However, after applying FDR correction, it was no longer significant (*p_FDR_* = 0.1290). The test for subgroup differences was not statistically significant (*Q* = 0.79, *p* = 0.3747).


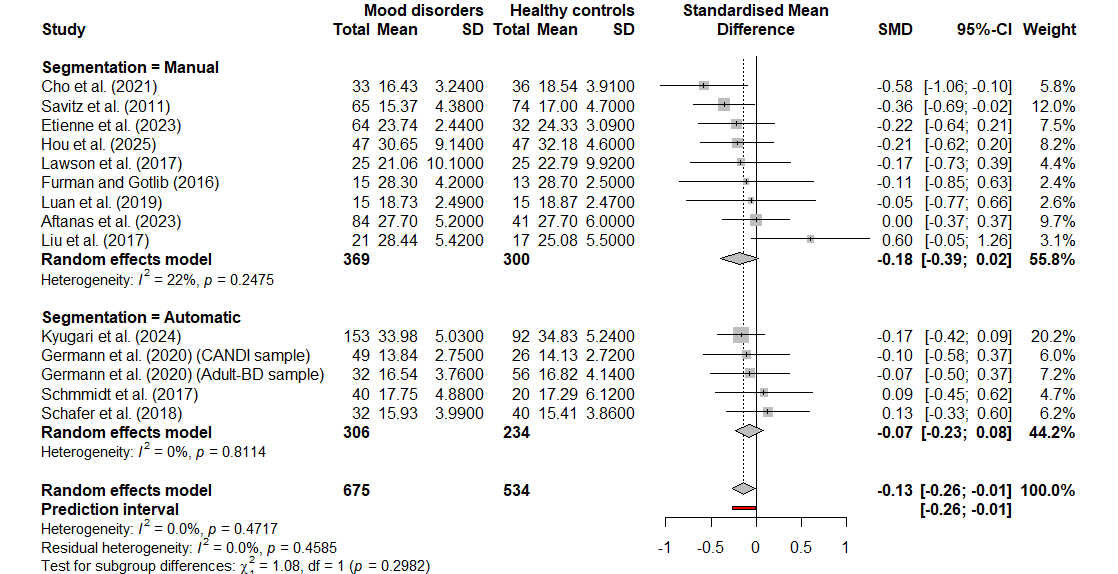


**Figure S8.** Forest plot presenting the subgroup analysis based on segmentation method for right habenula (individuals with a mood disorder versus HCs). Among samples using manual segmentation, there was no significant difference in habenula volume (*g* = -0.1837, 95% CI [-0.3910, 0.0237], *p* = 0.0754). Among samples using automatic segmentation, there was no significant difference in habenula volume (*g* = -0.0734, 95% CI [-0.2293, 0.0826], *p* = 0.2616). The test for subgroup differences was not statistically significant (*Q* = 1.08, *p* = 0.2982).


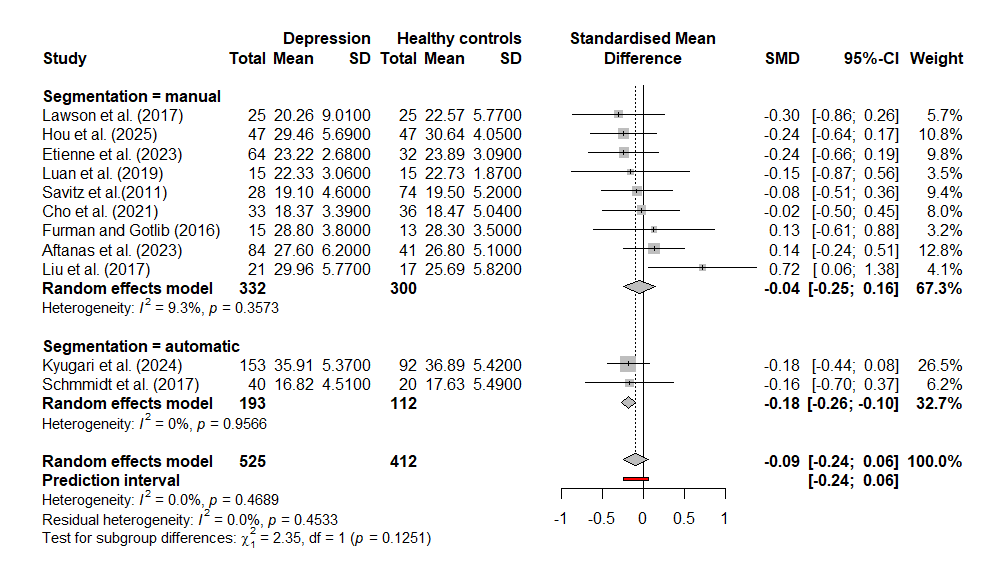
**Figure S9.** Forest plot presenting the subgroup analysis based on segmentation method for left habenula (individuals with MDD versus HCs). Among samples using manual segmentation, there was no significant difference in habenula volume (*g* = -0.0441, 95% CI [-0.2452, 0.1570], *p* = 0.6269). Among samples using automatic segmentation, the left habenula was significantly smaller in patients with MDD (*g* = -0.1782, 95% CI [-0.2604; -0.0959], *p* = 0.0231). However, after applying FDR correction, it was no longer significant (*p_FDR_* = 0.1540). The test for subgroup differences was not statistically significant (*Q* = 2.35, *p* = 0.1251).


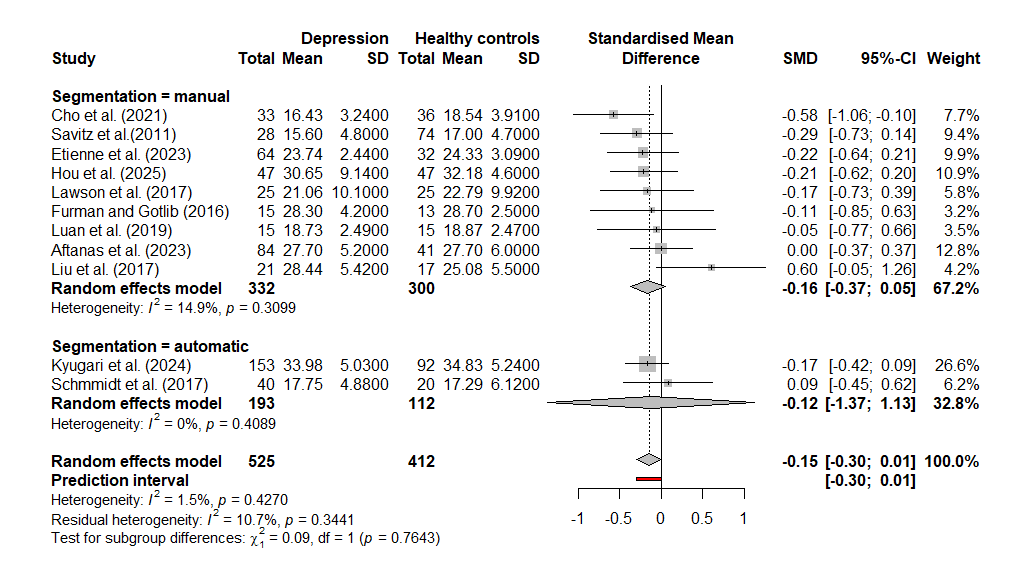
**Figure S10.** Forest plot presenting the subgroup analysis based on segmentation method for right habenula (individuals with MDD versus HCs). Among samples using manual segmentation, there was no significant difference in habenula volume (*g* =-0.1584, 95% CI [-0.3663, 0.0494], *p* = 0.1169). Among samples using automatic segmentation, there was no significant difference in habenula volume (*g* = -0.1184, 95% CI [-1.3674; 1.1306], *p* = 0.4410). The test for subgroup differences was not statistically significant (*Q* = 0.09, *p* = 0.7643).


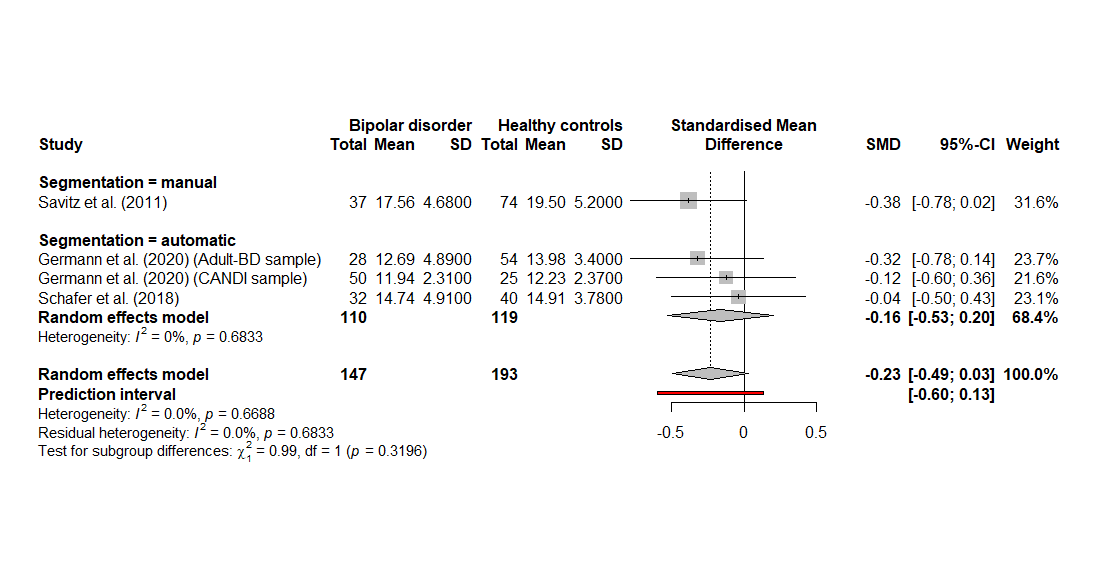
**Figure S11.** Forest plot presenting the subgroup analysis based on segmentation method for left habenula (individuals with BD versus HCs). Among samples using manual segmentation, there was no significant difference in habenula volume (*g* = -0.3827, 95% CI [-0.7806; 0.0152], *p* = 0.0594). Among samples using automatic segmentation, there was no significant difference in habenula volume (*g* = -0.1636, 95% CI [-0.5296, 0.2024], *p* = 0.1943). The test for subgroup differences was not statistically significant (*Q* = 0.99, *p* =0.3196).


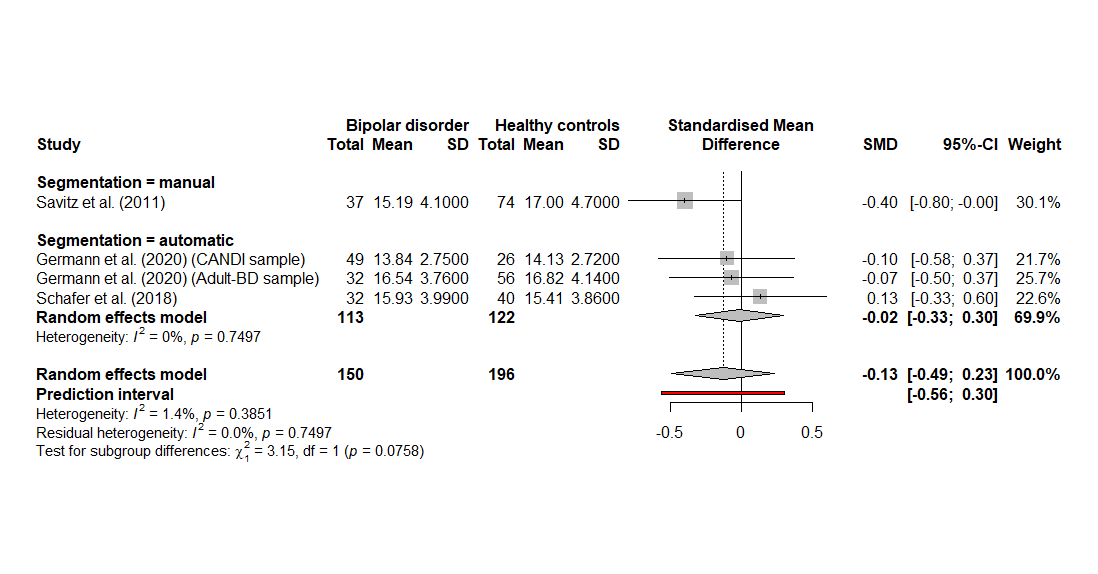
**Figure S12.** Forest plot presenting the subgroup analysis based on segmentation method for right habenula (individuals with BD versus HCs). Only one sample employed manual segmentation, which reported significantly smaller right habenula volume in patients with BD (g= -0.3985, 95% CI [-0.7967, -0.0003], *p* = 0.0498). However, after applying FDR correction, it was no longer significant (*p_FDR_* = 0.2846). Among samples using automatic segmentation, there was no significant difference in habenula volume (*g* = -0.0156, 95% CI [-0.3268, 0.2957], *p* = 0.8494). The test for subgroup differences was not statistically significant (*Q* = 3.15, *p* = 0.0758).

**Subgroup Analyses Based on Medication Status**


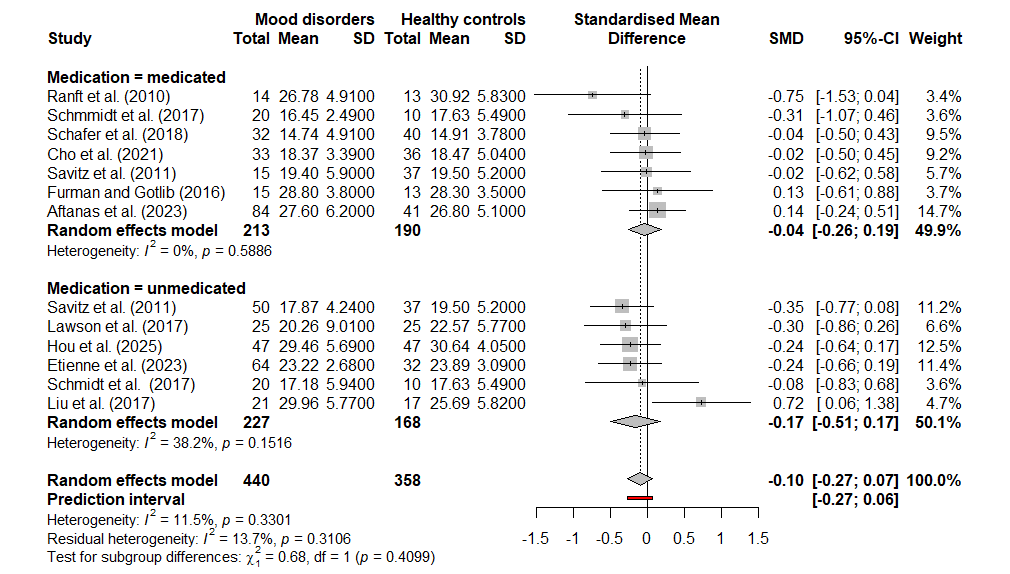


**Figure S13.** Forest plot presenting the subgroup analysis based on medication status for left habenula (individuals with a mood disorder versus HCs). Among medicated samples, there was no significant difference in habenula volume (*g* = -0.0357, 95% CI [-0.2594, 0.1879], *p* =0.7092). Among unmedicated samples, there was no significant difference in habenula volume (*g* = -0.1678, 95% CI [-0.5064, 0.1707], *p* = 0.2585). The test for subgroup differences was not statistically significant (*Q* = 0.68, *p* = 0.4099).


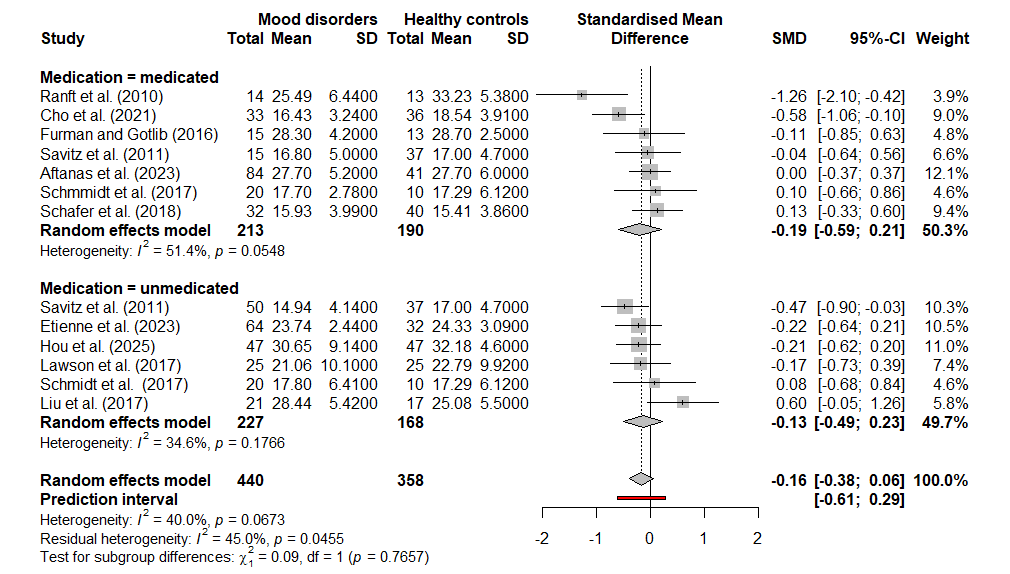


**Figure S14.** Forest plot presenting the subgroup analysis based on medication status for right habenula (individuals with a mood disorder versus HCs). Among medicated samples, there was no significant difference in habenula volume (*g* = -0.1914, 95% CI [-0.5932, 0.2105], *p* = 0.2881). Among unmedicated samples, there was no significant difference in habenula volume (*g* = -0.1272, 95% CI [-0.4856, 0.2312], *p* = 0.4035). The test for subgroup differences was not statistically significant (*Q* = 0.09, *p* = 0.7657).


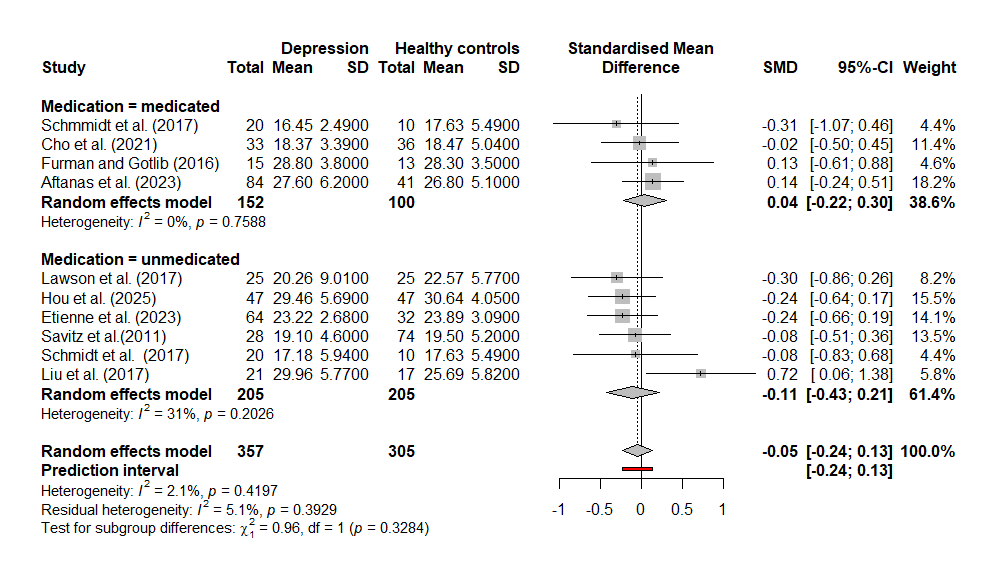


**Figure S15.** Forest plot presenting the subgroup analysis based on medication status for left habenula (individuals with MDD versus HCs). Among medicated samples, there was no significant difference in habenula volume (*g* = 0.0382, 95% CI [-0.2186, 0.2950], *p* = 0.6731). Among unmedicated samples, there was no significant difference in habenula volume (*g* = -0.1080, 95% CI [-0.3117, 0.0956], *p* = 0.4275). The test for subgroup differences was not statistically significant (*Q* = 0.96, *p* = 0.3284).


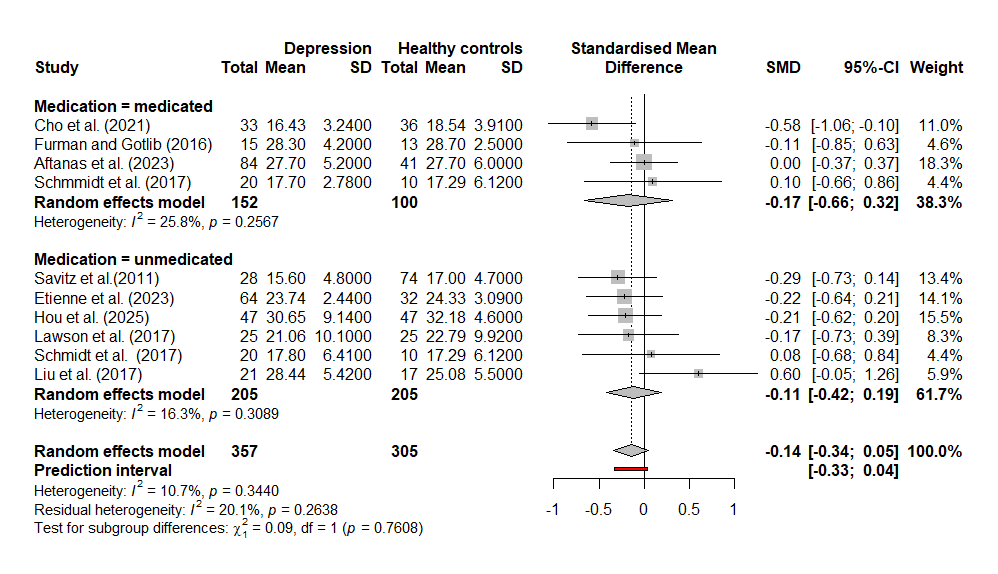


**Figure S16.** Forest plot presenting the subgroup analysis based on medication status for right habenula (individuals with MDD versus HCs). Among medicated samples, there was no significant difference in habenula volume (*g* = -0.1722, 95% CI [-0.6633, 0.3190], *p* = 0.3459). Among unmedicated samples, there was no significant difference in habenula volume (*g* = -0.1130, 95% CI [-0.4173, 0.1914], *p* = 0.3838). The test for subgroup differences was not statistically significant (*Q* = 0.09, *p* = 0.7608).


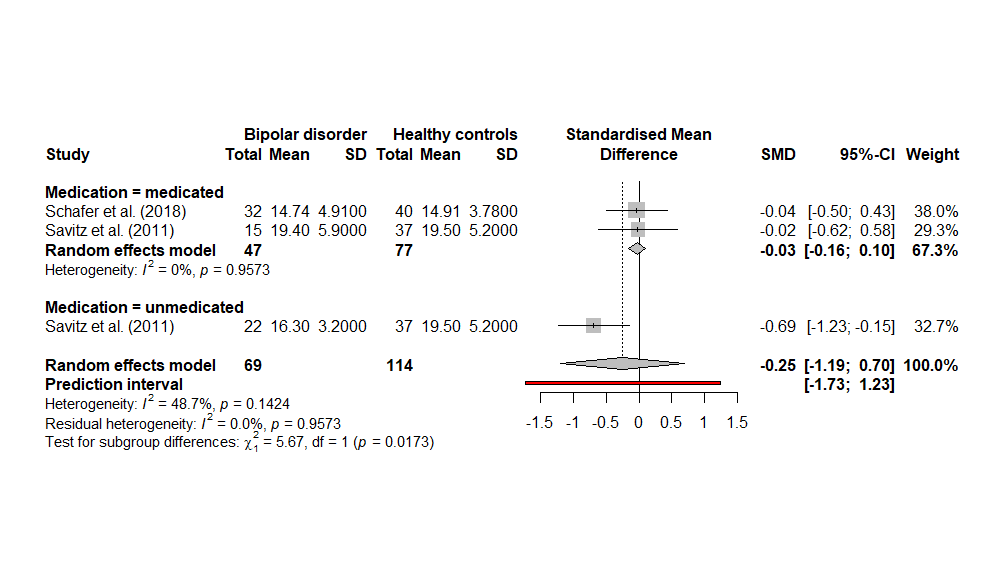


**Figure S17.** Forest plot presenting the subgroup analysis based on medication status for left habenula (individuals with BD versus HCs). Among medicated samples, there was no significant difference in habenula volume (*g* = -0.0312, 95% CI [-0.1587, 0.0964], *p* = 0.1983). Only one sample reported data from unmedicated patients, which reported significantly smaller left habenula volume in patients with BD (*g* = -0.6915, 95% CI [-1.2347, -0.1483], *p* = 0.0126). However, after applying FDR correction, it was no longer significant (*p_FDR_* = 0.1290). The test for subgroup differences was statistically significant, indicating that the effect size in the unmedicated subgroup was significantly more negative than in the medicated subgroup (*Q* = 5.67, *p* = 0.0173). However, after applying FDR correction, it was no longer significant (*p_FDR_* = 0.1384).


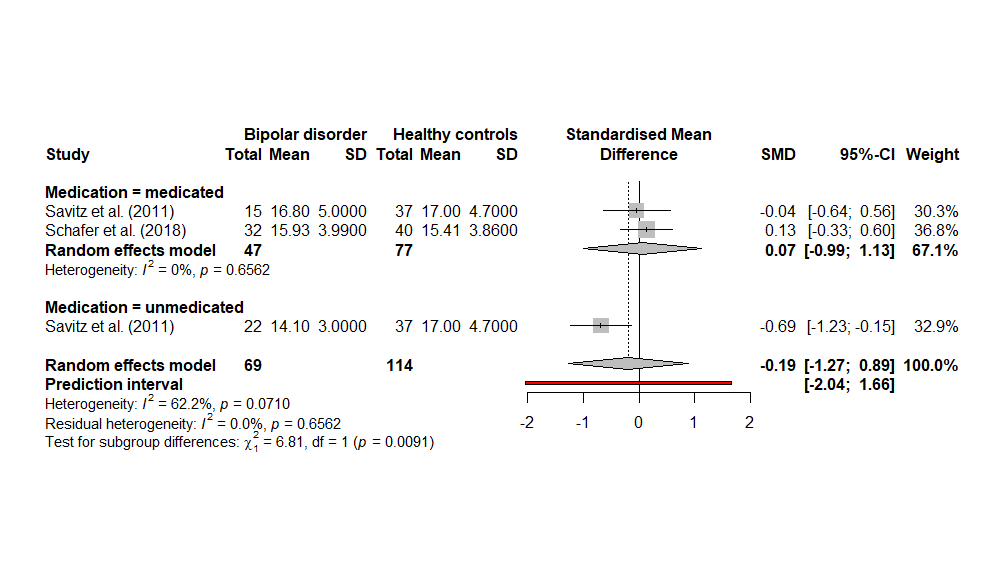


**Figure S18.** Forest plot presenting the subgroup analysis based on medication status for right habenula (individuals with BD versus HCs). Among medicated samples, there was no significant difference in habenula volume (*g* = 0.0665, 95% CI [-0.9946, 1.1277], *p* = 0.5718). Only one sample reported data from unmedicated patients, which reported significantly smaller left habenula volume in patients with BD (*g* = -0.6887, 95% CI [-1.2317, -0.1456], *p* = 0.0129). However, after applying FDR correction, it was no longer significant (*p_FDR_* = 0.1290). The test for subgroup differences was statistically significant, indicating that the effect size in the unmedicated subgroup was significantly more negative than in the medicated subgroup (*Q* = 6.81, *p* = 0.0091). However, after applying FDR correction, it was no longer significant (*p_FDR_* = 0.1290).

**Sensitivity Analyses**

As specified in the registration, because *I²* was below 50%, we did not check for outliers or conduct an influential analysis. We nevertheless conducted leave-one-out analyses.

We also conducted sensitivity in which we varied the estimated standard deviations for Ranft et al. (10). Indeed, in this study, total habenula volume and its standard deviation were not directly provided. We also conducted sensitivity analyses in which we varied the estimated standard deviations reported in Ranft et al. (11), as total habenula volume and its standard deviation were not directly provided. Instead, the total volume was derived by summing the medial and lateral subregion volumes, and the standard deviation was estimated using Formula 2, which requires assuming a correlation coefficient (*r*) between these subregions. As no relevant estimates for this correlation were identified in the literature, we conducted our primary analyses assuming *r* = 0.5. To evaluate the impact of this assumption on our findings, we conducted sensitivity analyses using alternative values of *r* = 1 and *r* = 0.

For the left habenula, assuming r = 1, we observed a small but statistically significant reduction in volume among individuals with a mood disorder compared to HCs (*g* = –0.1357, 95% CI [–0.2590, –0.0123], *p* = .0334). Similarly, under the assumption of r = 0, the effect remained significant (*g* = –0.1381, 95% CI [–0.2656, –0.0105], *p* = .0359). Overall, these results suggest that our findings are robust to reasonable variations in the imputed correlation coefficient used for the Ranft et al. (10) study.

For the right habenula, assuming r = 1 between the two subregion volumes, we observed a small but statistically significant reduction in volume among individuals with a mood disorder compared to HCs (*g* = -0.1551, 95% CI [-0.3007; -0.0095], *p* = 0.0384). Similarly, under the assumption of r = 0, the effect remained significant (*g* = -0.1574, 95% CI [-0.3100; -0.0048], *p* = 0.0441). Overall, these results suggest that our findings are robust to reasonable variations in the imputed correlation coefficient used for the Ranft et al. (10) study.

**Leave-One-Out Analyses**


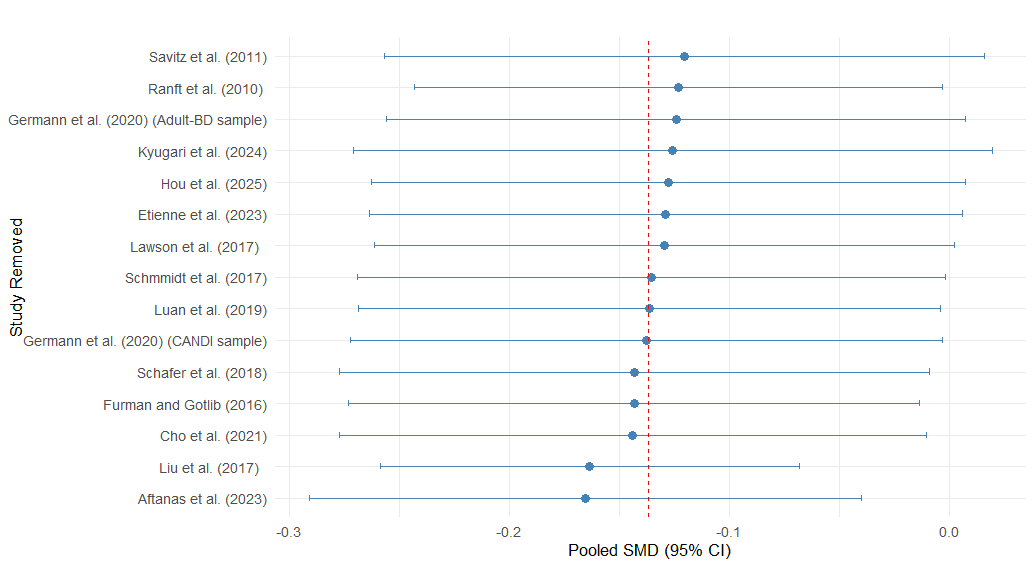


**Figure S19.** Plot presenting the results of the leave-one-out analysis for left habenula (individuals with a mood disorder versus HCs).

**
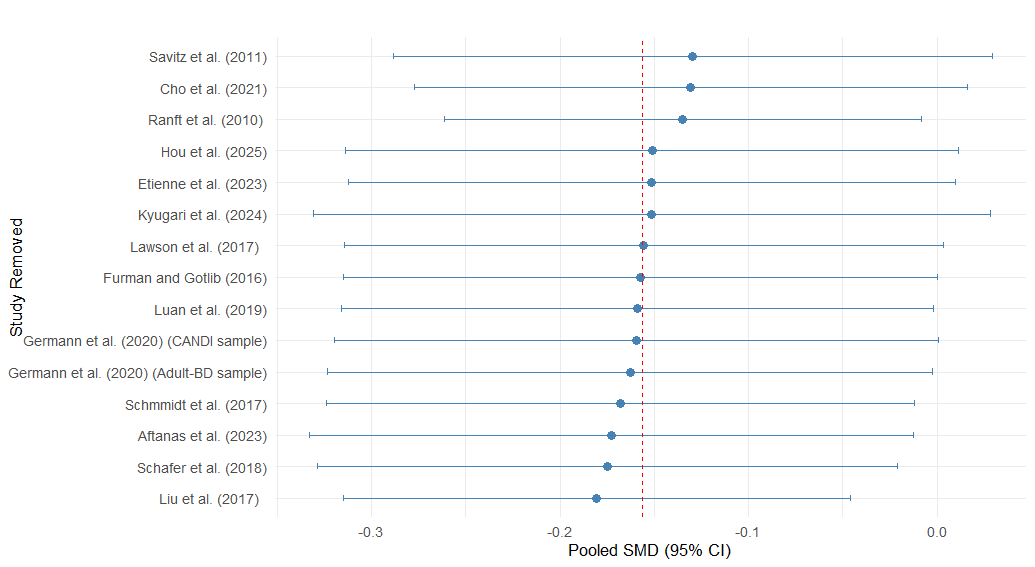
**

**Figure S20.** Plot presenting the results of the leave-one-out analysis for right habenula (individuals with a mood disorder versus HCs).

**
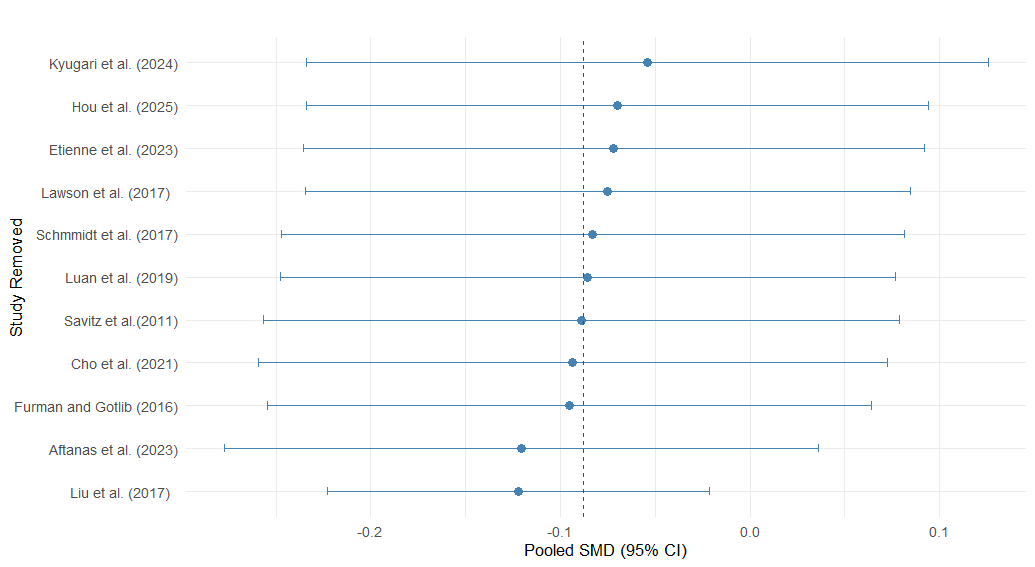
**

**Figure S21.** Plot presenting the results of the leave-one-out analysis for left habenula (individuals with MDD versus HCs).

**
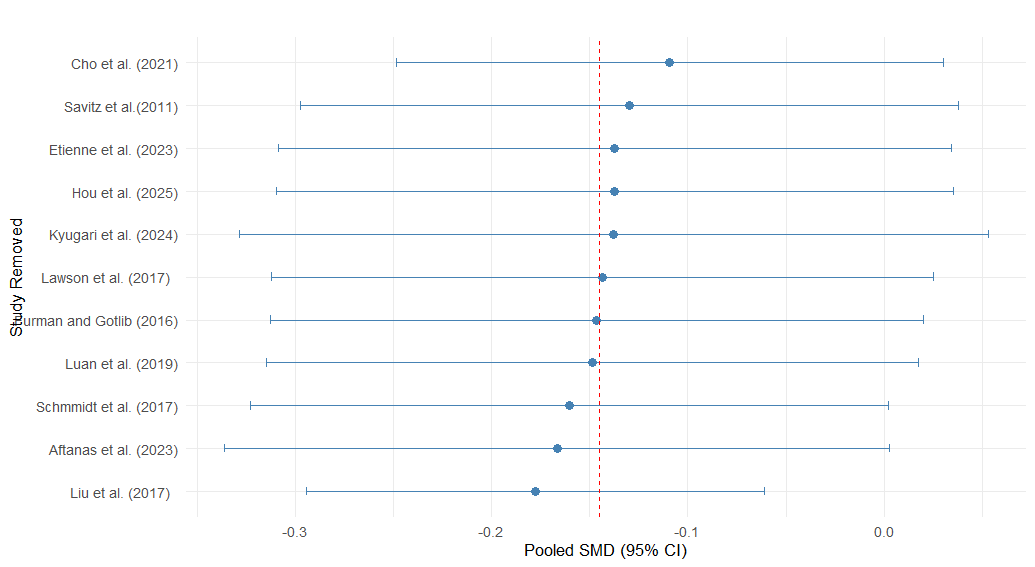
**

**Figure S22.** Plot presenting the results of the leave-one-out analysis for right habenula (individuals with MDD versus HCs).


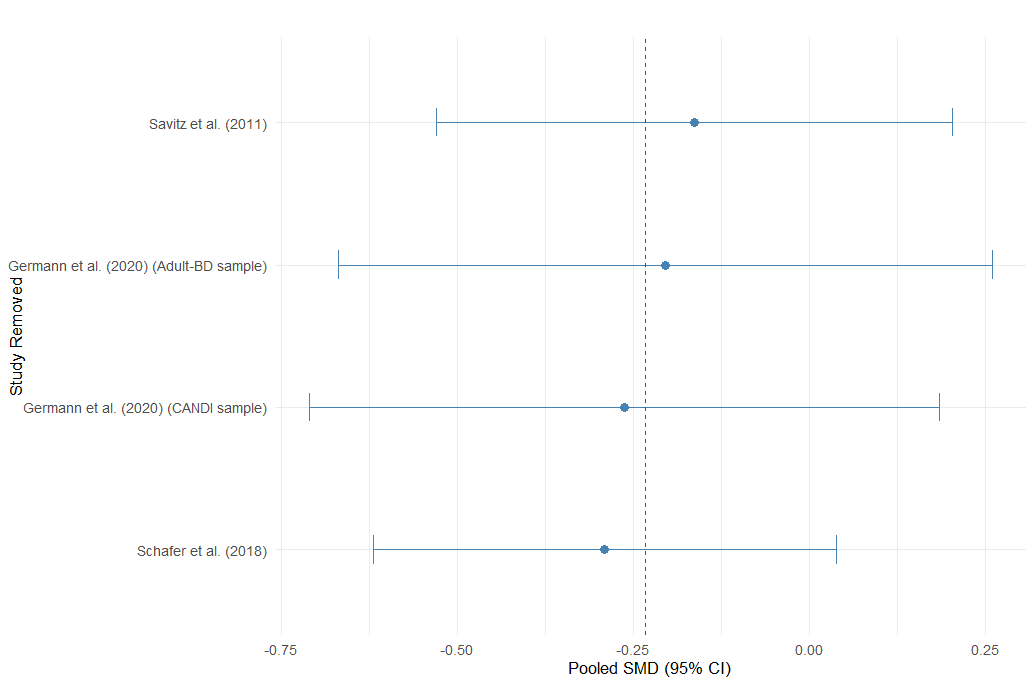


**Figure S23.** Plot presenting the results of the leave-one-out analysis for left habenula (individuals with BD versus HCs).

**
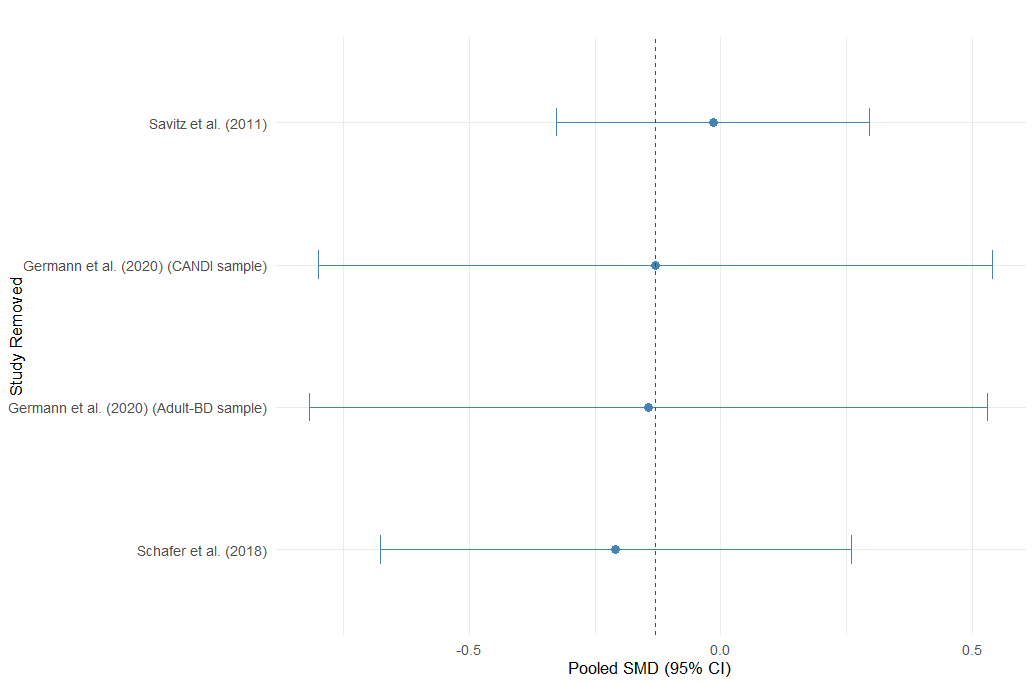
**

**Figure S24.** Plot presenting the results of the leave-one-out analysis for right habenula (individuals with BD versus HCs)

**Funnel Plots**
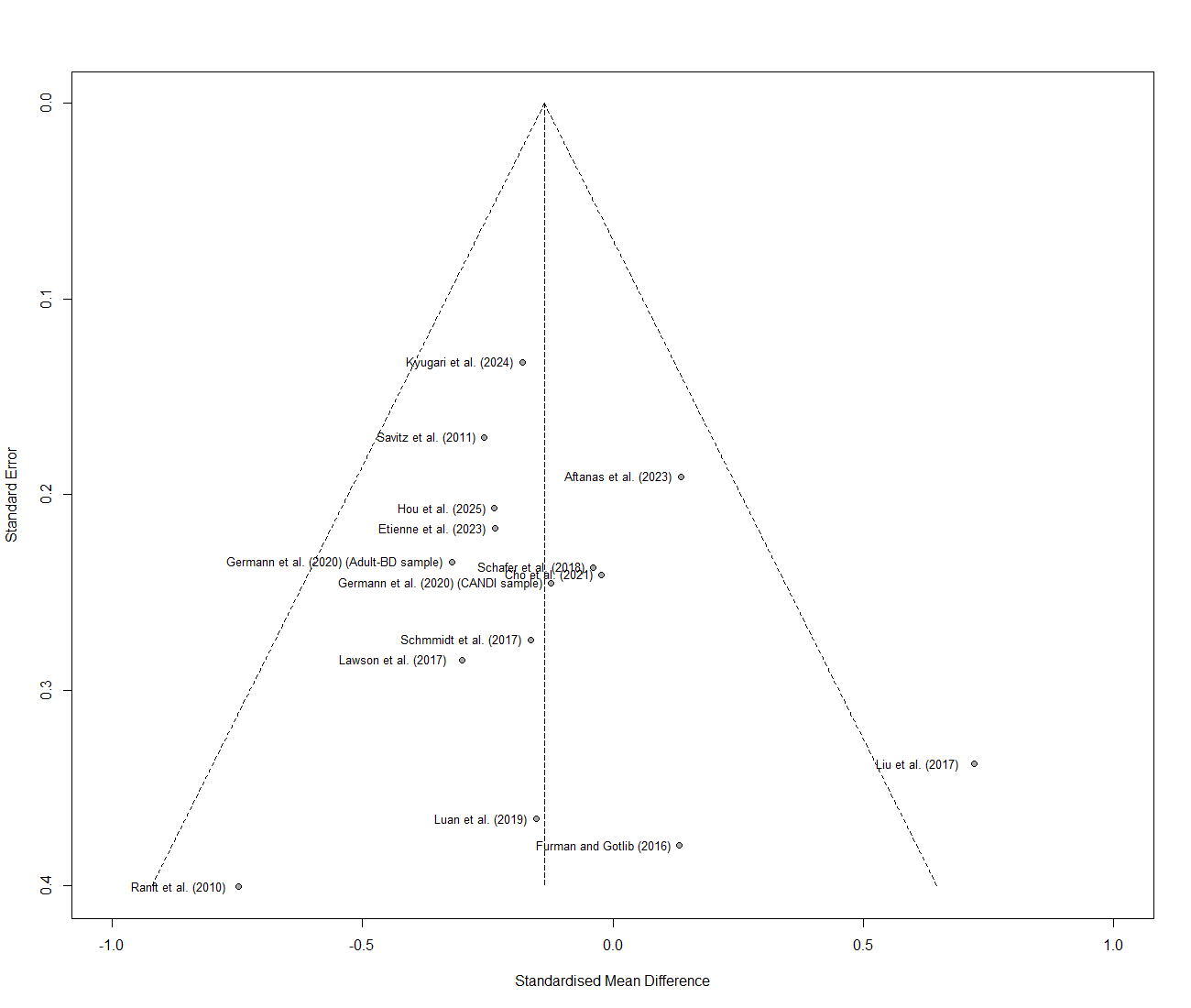


**Figure S25.** Funnel plot of left habenula (individuals with a mood disorder versus HCs).


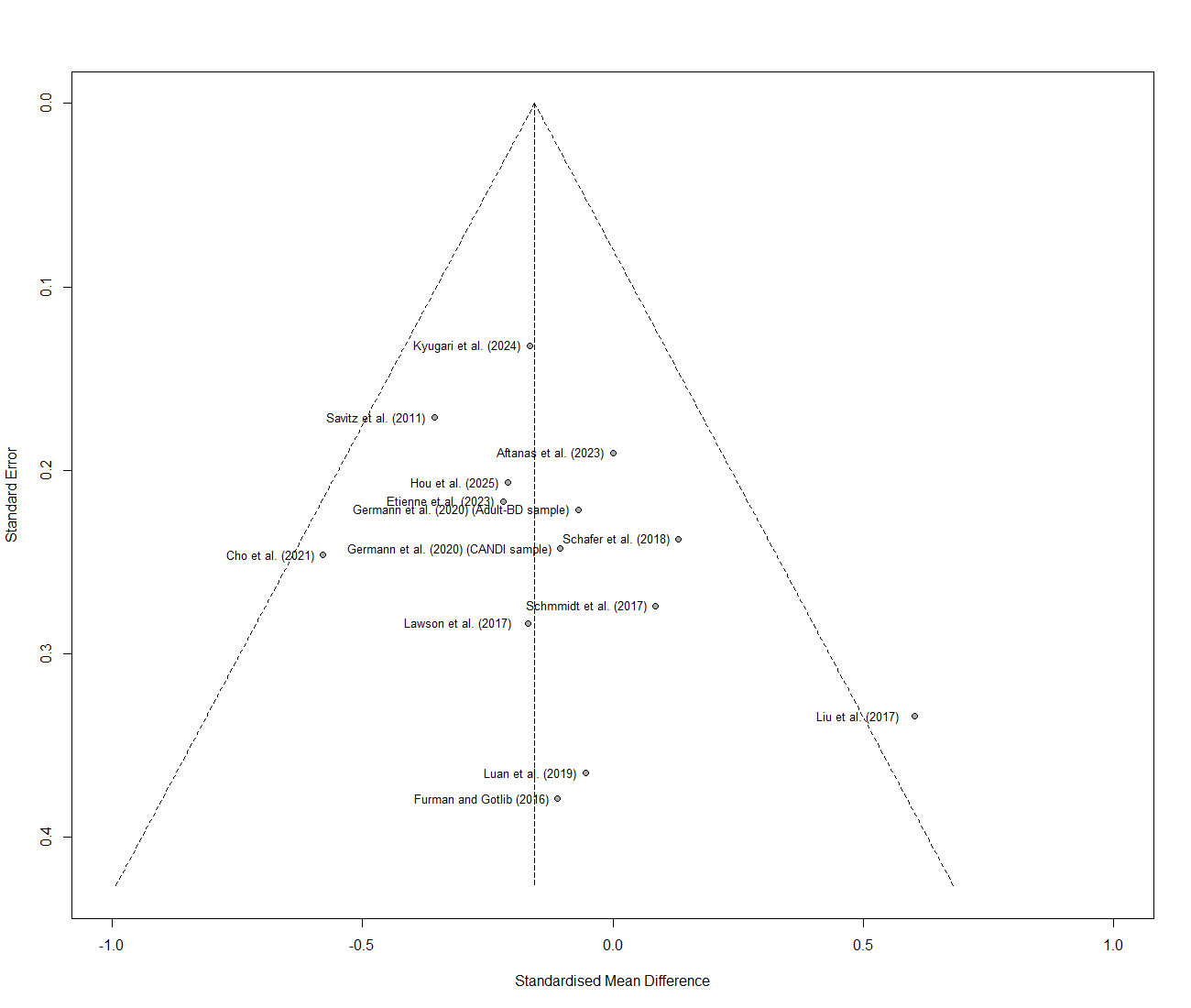


**Figure S26.** Funnel plot of right habenula volume (individuals with a mood disorder versus HCs).


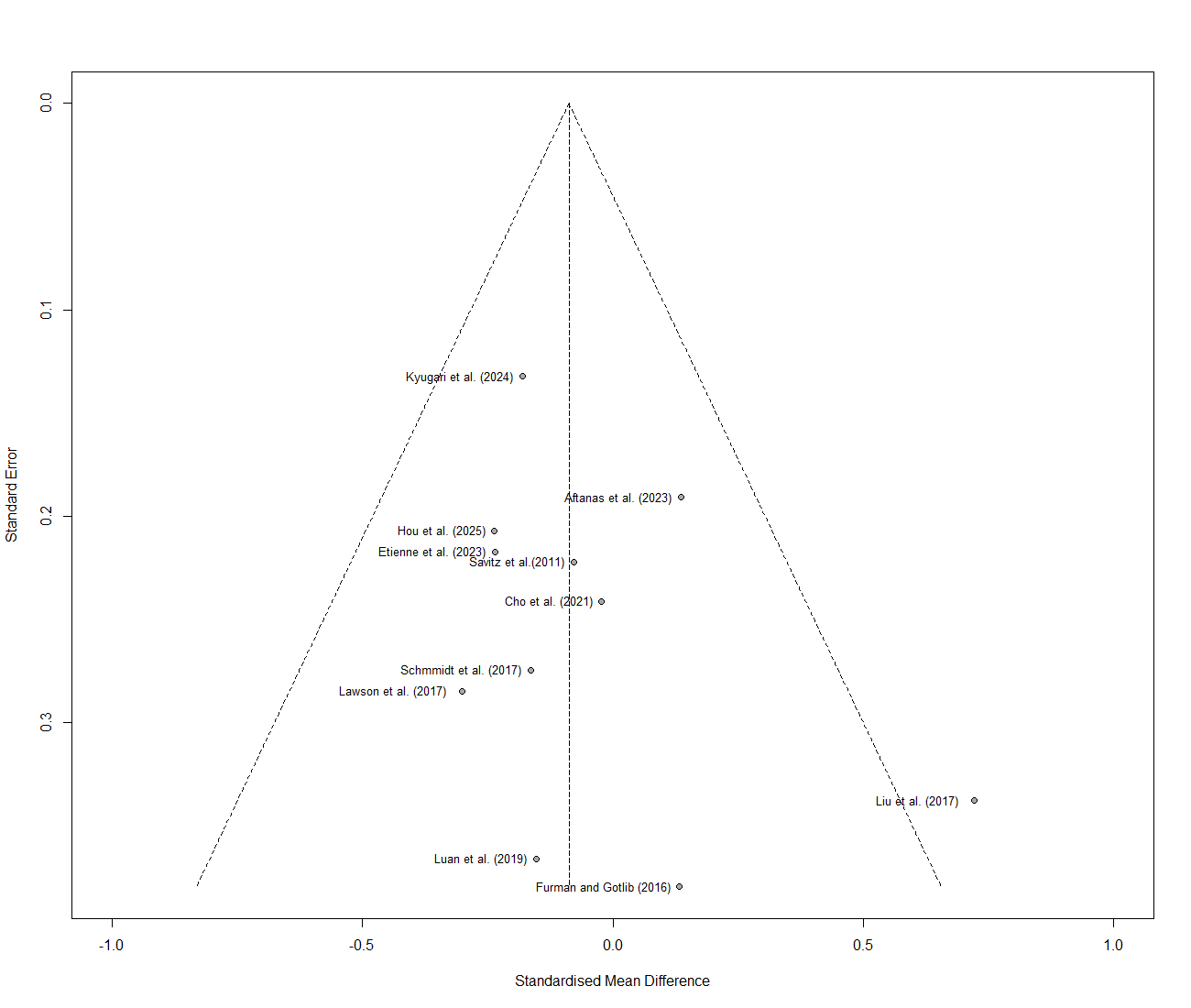


**Figure S27.** Funnel plot of left habenula (individuals with MDD versus HCs).


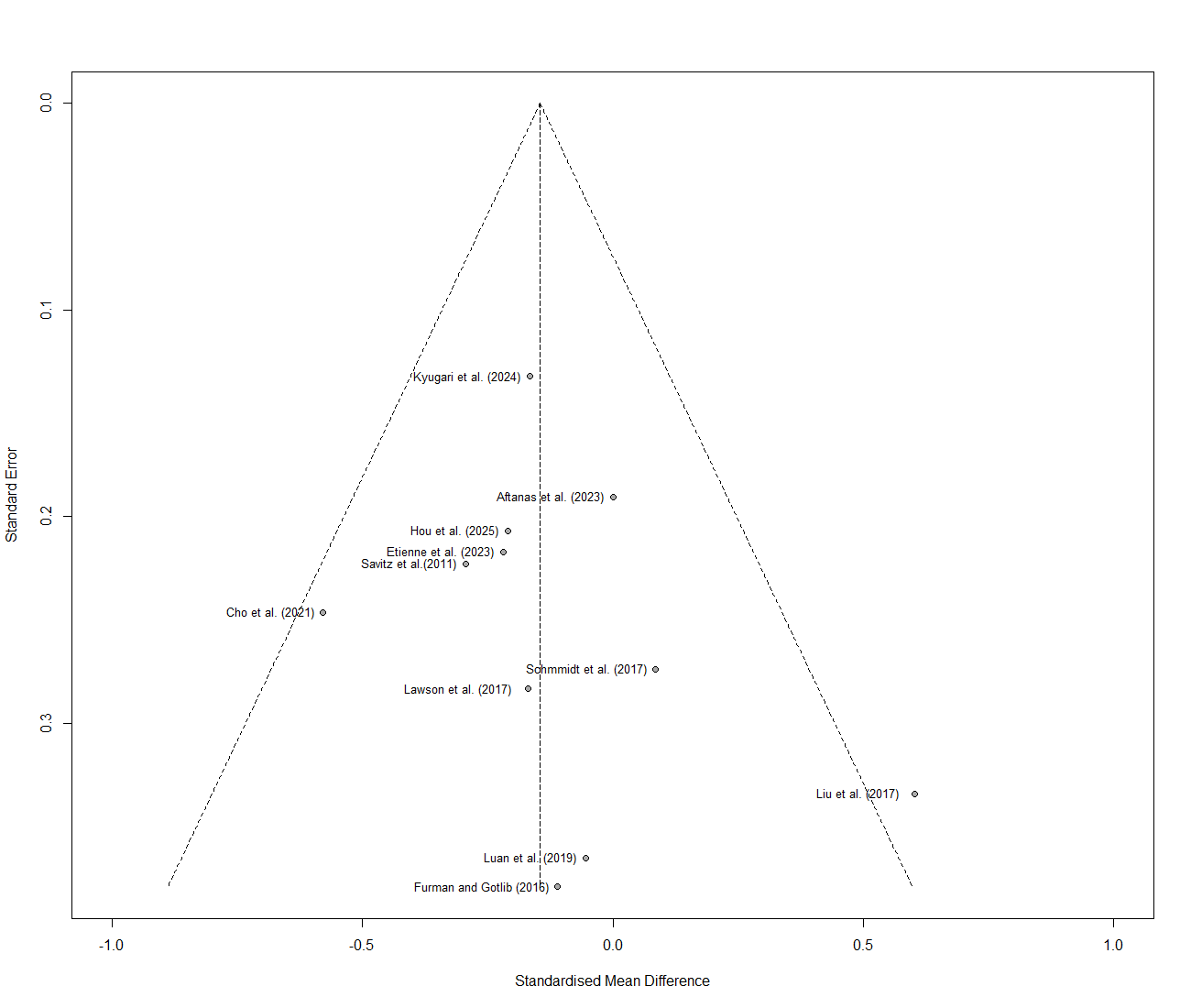


**Figure S28.** Funnel plot of right habenula (individuals with MDD versus HCs).

| **Table S1.** Quality assessment of studies with the NIH Quality Assessment Tool for Observational Cohort and Cross-Sectional Studies (21) | | | | | | | | | | | | | | |  |
| --- | --- | --- | --- | --- | --- | --- | --- | --- | --- | --- | --- | --- | --- | --- | --- |
| **Authors** | 1 | 2 | 3 | 4 | 5 | 6 | 7 | 8 | 9 | 10 | 11 | 12 | 13 | 14 | Quality rating |
| Aftanas et al. (22) | Y | Y | NR | Y | N | N | N | N | Y | N | Y | NR | NA | NA | Fair |
| Cho et al. (4) | Y | Y | NR | NR | N | N | N | N | Y | N | Y | Y | NA | NA | Fair |
| Etienne et al. (23) | Y | Y | NR | NR | Y | N | N | N | Y | N | Y | Y | NA | NA | Good |
| Furman et Gotlib (24) | Y | Y | NR | NR | N | N | N | N | Y | N | Y | NR | NA | NA | Fair |
| Germann et al.  CANDI sample (7) | Y | N | NR | NR | N | N | N | N | Y | N | Y | NR | NA | NA | Poor |
| Germann et al.  Adult-BD sample (7) | Y | Y | NR | NR | N | N | N | N | Y | N | Y | NR | NA | NA | Fair |
| Hou et al. (25) | Y | N | NR | NR | N | N | N | N | Y | N | Y | NR | NA | NA | Poor |
| Kyugari et al. (26) | Y | Y | NR | Y | N | N | N | N | Y | N | Y | Y | NA | NA | Good |
| Lawson et al. (8) | Y | Y | NR | N | N | N | N | N | Y | N | Y | NR | NA | NA | Fair |
| Liu et al. (9) | Y | Y | NR | N | N | N | N | N | Y | N | Y | NR | NA | NA | Fair |
| Luan et al. (27) | Y | Y | NR | N | N | N | N | N | Y | N | Y | NR | NA | NA | Fair |
| Ranft et al. (10) | Y | Y | NA | Y | N | N | N | N | N | N | Y | Y | NA | NA | Fair |
| Savitz et al. (11) | Y | N | NR | NR | N | N | N | Y | Y | N | Y | Y | NA | NA | Fair |
| Schafer et al. (28) | Y | Y | NR | Y | N | N | N | N | Y | N | Y | Y | NA | NA | Good |
| Schmidt et al. (12) | Y | N | NR | NR | N | N | N | Y | Y | N | Y | Y | NA | NA | Fair |

*1* Was the research question or objective in this paper clearly stated? *2* Was the study population clearly specified and defined? *3* Was the participation rate of eligible persons at least 50%? *4* Were all the subjects selected or recruited from the same or similar populations (including the same time period)? Were inclusion and exclusion criteria for being in the study prespecified and applied uniformly to all participants? *5* Was a sample size justification, power description, or variance and effect estimates provided? *6* For the analyses in this paper, were the exposure(s) of interest measured prior to the outcome(s) being measured? *7* Was the timeframe sufficient so that one could reasonably expect to see an association between exposure and outcome if it existed? *8* For exposures that can vary in amount or level, did the study examine different levels of the exposure as related to the outcome (e.g., categories of exposure, or exposure measured as continuous variable)? *9* Were the exposure measures (independent variables) clearly defined, valid, reliable, and implemented consistently across all study participants? *10* Was the exposure(s) assessed more than once over time? *11* Were the outcome measures (dependent variables) clearly defined, valid, reliable, and implemented consistently across all study participants? *12* Were the outcome assessors blinded to the exposure status of participants? *13* Was loss to follow-up after baseline 20% or less? *14* Were key potential confounding variables measured and adjusted statistically for their impact on the relationship between exposure(s) and outcome(s)? *CD* cannot determine, *NA* not applicable, *NR* not reported

**Table S2.** Studies excluded during full-text screening and the reason for their exclusion.

| **#** | **Study** | **Reason for Exclusion** |
| --- | --- | --- |
| 1 | Amiri et al. (29) | No volumetric results reported |
| 2 | Carceller-Sindreu et al. (30) | No volumetric results reported |
| 3 | Aftanas et al. (31) | Conference abstract |
| 4 | Gao et al. (32) | No volumetric results reported |
| 5 | Sartorius et al. (33) | No volumetric results reported |
| 6 | Etienne et al. (34) | No control group |
| 7 | Liu et al. (9) | Conference abstract |
| 8 | Ely et al. (35) | No volumetric results reported |
| 9 | Kumar et al. (36) | No volumetric results reported |
| 10 | Savitz et al. (37) | No mood disorder component |
| 11 | Sartorius et al. (38) | No volumetric results reported |
| 12 | Dai et al. (39) | No mood disorder component |
| 13 | Samanci et al. (40) | No mood disorder component |
| 14 | Elias et al. (41) | No control group |
| 15 | Johnston et al. (42) | No volumetric results reported |
| 16 | Jung et al. (43) | No volumetric results reported |
| 17 | Anand et al. (44) | No volumetric results reported |
| 18 | de Diego-Adeliño et al. (45) | Conference abstract |
| 19 | Salas et al. (46) | Conference abstract |

If a study met multiple exclusion criteria, we applied them in the following priority order: conference abstract, no mood disorder component, no volumetric results reported, no control group.

**PRISMA 2020 Checklist**

| **Section and Topic** | **Item #** | **Checklist item** | **Location where item is reported** |
| --- | --- | --- | --- |
| **TITLE** | | |  |
| Title | 1 | Identify the report as a systematic review | NA |
| **ABSTRACT** | | |  |
| Abstract | 2 | See the PRISMA 2020 for Abstracts checklist. | p.2 |
| **INTRODUCTION** | | |  |
| Rationale | 3 | Describe the rationale for the review in the context of existing knowledge. | p.5 |
| Objectives | 4 | Provide an explicit statement of the objective(s) or question(s) the review addresses. | p.5 |
| **METHODS** | | |  |
| Eligibility criteria | 5 | Specify the inclusion and exclusion criteria for the review and how studies were grouped for the syntheses. | p.6 |
| Information sources | 6 | Specify all databases, registers, websites, organisations, reference lists and other sources searched or consulted to identify studies. Specify the date when each source was last searched or consulted. | p.6 |
| Search strategy | 7 | Present the full search strategies for all databases, registers and websites, including any filters and limits used. | Supplemental Methods and Materials |
| Selection process | 8 | Specify the methods used to decide whether a study met the inclusion criteria of the review, including how many reviewers screened each record and each report retrieved, whether they worked independently, and if applicable, details of automation tools used in the process. | p.6 |
| Data collection process | 9 | Specify the methods used to collect data from reports, including how many reviewers collected data from each report, whether they worked independently, any processes for obtaining or confirming data from study investigators, and if applicable, details of automation tools used in the process. | p.7 |
| Data items | 10a | List and define all outcomes for which data were sought. Specify whether all results that were compatible with each outcome domain in each study were sought (e.g. for all measures, time points, analyses), and if not, the methods used to decide which results to collect. | Supplemental Methods and Materials |
|  | 10b | List and define all other variables for which data were sought (e.g. participant and intervention characteristics, funding sources). Describe any assumptions made about any missing or unclear information. | Supplemental Methods and Materials |
| Study risk of bias assessment | 11 | Specify the methods used to assess risk of bias in the included studies, including details of the tool(s) used, how many reviewers assessed each study and whether they worked independently, and if applicable, details of automation tools used in the process. | p.7 and Supplemental Methods and Materials |
| Effect measures | 12 | Specify for each outcome the effect measure(s) (e.g. risk ratio, mean difference) used in the synthesis or presentation of results. | p.8 |
| Synthesis methods | 13a | Describe the processes used to decide which studies were eligible for each synthesis (e.g. tabulating the study intervention characteristics and comparing against the planned groups for each synthesis (item #5)). | p.7 |
|  | 13b | Describe any methods required to prepare the data for presentation or synthesis, such as handling of missing summary statistics, or data conversions. | Supplemental Methods and Materials |
|  | 13c | Describe any methods used to tabulate or visually display results of individual studies and syntheses. | Supplemental Methods and Materials |
|  | 13d | Describe any methods used to synthesize results and provide a rationale for the choice(s). If meta-analysis was performed, describe the model(s), method(s) to identify the presence and extent of statistical heterogeneity, and software package(s) used. | p.7-8 and Supplemental Methods and Materials |
|  | 13e | Describe any methods used to explore possible causes of heterogeneity among study results (e.g. subgroup analysis, meta-regression). | Supplemental Methods and Materials |
|  | 13f | Describe any sensitivity analyses conducted to assess robustness of the synthesized results. | Supplemental Methods and Materials |
| Reporting bias assessment | 14 | Describe any methods used to assess risk of bias due to missing results in a synthesis (arising from reporting biases). | Supplemental Methods and Materials |
| Certainty assessment | 15 | Describe any methods used to assess certainty (or confidence) in the body of evidence for an outcome. | NA |
| **RESULTS** | | |  |
| Study selection | 16a | Describe the results of the search and selection process, from the number of records identified in the search to the number of studies included in the review, ideally using a flow diagram. | p. 6-7 |
|  | 16b | Cite studies that might appear to meet the inclusion criteria, but which were excluded, and explain why they were excluded. | NA |
| Study characteristics | 17 | Cite each included study and present its characteristics. | Table 1 |
| Risk of bias in studies | 18 | Present assessments of risk of bias for each included study. | Table S1 |
| Results of individual studies | 19 | For all outcomes, present, for each study: (a) summary statistics for each group (where appropriate) and (b) an effect estimate and its precision (e.g. confidence/credible interval), ideally using structured tables or plots. | Figure 2 and Figure 3 |
| Results of syntheses | 20a | For each synthesis, briefly summarise the characteristics and risk of bias among contributing studies. | NA |
|  | 20b | Present results of all statistical syntheses conducted. If meta-analysis was done, present for each the summary estimate and its precision (e.g. confidence/credible interval) and measures of statistical heterogeneity. If comparing groups, describe the direction of the effect. | p.9-10 and Supplemental Results |
|  | 20c | Present results of all investigations of possible causes of heterogeneity among study results. | p.9-10 and Supplemental Results |
|  | 20d | Present results of all sensitivity analyses conducted to assess the robustness of the synthesized results. | p.9-10 and Supplemental Results |
| Reporting biases | 21 | Present assessments of risk of bias due to missing results (arising from reporting biases) for each synthesis assessed. | p.9-10 and Supplemental Results |
| Certainty of evidence | 22 | Present assessments of certainty (or confidence) in the body of evidence for each outcome assessed. | NA |
| **DISCUSSION** | | |  |
| Discussion | 23a | Provide a general interpretation of the results in the context of other evidence. | p.11-15 |
|  | 23b | Discuss any limitations of the evidence included in the review. | p.15-16 |
|  | 23c | Discuss any limitations of the review processes used. | p.15-16 |
|  | 23d | Discuss implications of the results for practice, policy, and future research. | p.15-16 |
| **OTHER INFORMATION** | | |  |
| Registration and protocol | 24a | Provide registration information for the review, including register name and registration number, or state that the review was not registered. | p.6 |
|  | 24b | Indicate where the review protocol can be accessed, or state that a protocol was not prepared. | p.6 |
|  | 24c | Describe and explain any amendments to information provided at registration or in the protocol. | Supplemental Information p.1 |
| Support | 25 | Describe sources of financial or non-financial support for the review, and the role of the funders or sponsors in the review. | p.17 |
| Competing interests | 26 | Declare any competing interests of review authors. | p.17 |
| Availability of data, code and other materials | 27 | Report which of the following are publicly available and where they can be found: template data collection forms; data extracted from included studies; data used for all analyses; analytic code; any other materials used in the review. | p.17 |

**References**

1. Borenstein M, Hedges LV, Higgins JPT, Rothstein HR (2011): *Introduction to Meta-Analysis*. Chichester, UK: John Wiley & Sons.

2. Bramer WM, Rethlefsen ML, Kleijnen J, Franco OH (2017): Optimal database combinations for literature searches in systematic reviews: a prospective exploratory study. *Systematic Reviews*. 6:245.

3. Lim S-H, Yoon J, Kim YJ, Kang C-K, Cho S-E, Kim KG, Kang S-G (2021): Reproducibility of automated habenula segmentation via deep learning in major depressive disorder and normal controls with 7 Tesla MRI. *Scientific Reports*. 11:13445.

4. Cho SE, Park CA, Na KS, Chung C, Ma HJ, Kang CK, Kang SG (2021): Left-right asymmetric and smaller right habenula volume in major depressive disorder on high-resolution 7-T magnetic resonance imaging. *PLoS One*. 16:e0255459.

5. Landis JR, Koch GG (1977): The measurement of observer agreement for categorical data. *Biometrics*. 33:159-174.

6. Rohatgi A ( ): WebPlotDigitizer. 5.2 ed.

7. Germann J, Gouveia FV, Martinez RCR, Zanetti MV, de Souza Duran FL, Chaim-Avancini TM, et al. (2020): Fully Automated Habenula Segmentation Provides Robust and Reliable Volume Estimation Across Large Magnetic Resonance Imaging Datasets, Suggesting Intriguing Developmental Trajectories in Psychiatric Disease. *Biological Psychiatry: Cognitive Neuroscience and Neuroimaging*. 5:923-929.

8. Lawson RP, Nord CL, Seymour B, Thomas DL, Dayan P, Pilling S, Roiser JP (2017): Disrupted habenula function in major depression. *Mol Psychiatry*. 22:202-208.

9. Liu WH, Valton V, Wang LZ, Zhu YH, Roiser JP (2017): Association between habenula dysfunction and motivational symptoms in unmedicated major depressive disorder. *Soc Cogn Affect Neurosci*. 12:1520-1533.

10. Ranft K, Dobrowolny H, Krell D, Bielau H, Bogerts B, Bernstein HG (2010): Evidence for structural abnormalities of the human habenular complex in affective disorders but not in schizophrenia. *Psychological Medicine*. 40:557-567.

11. Savitz JB, Nugent AC, Bogers W, Roiser JP, Bain EE, Neumeister A, et al. (2011): Habenula Volume in Bipolar Disorder and Major Depressive Disorder: A High-Resolution Magnetic Resonance Imaging Study. *Biological Psychiatry*. 69:336-343.

12. Schmidt FM, Schindler S, Adamidis M, Strauß M, Tränkner A, Trampel R, et al. (2017): Habenula volume increases with disease severity in unmedicated major depressive disorder as revealed by 7T MRI. *Eur Arch Psychiatry Clin Neurosci*. 267:107-115.

13. Higgins JPTT, J.; Chandler, J.; Cumpston, M.; Li, T.; Page, M.J.; Welch, V.A. (2024): Cochrane Handbook for Systematic Reviews of Interventions version 6.5 (updated August 2024). Cochrane.

14. Borenstein M, Hedges LV, Higgins JP, Rothstein HR (2021): *Introduction to meta-analysis*. John wiley & sons.

15. Harrer M, Cuijpers P, Furukawa T, Ebert DD (2019): dmetar: Companion R Package For The Guide 'Doing Meta-Analysis in R.

16. Viechtbauer W (2005): Bias and Efficiency of Meta-Analytic Variance Estimators in the Random-Effects Model. *Journal of Educational and Behavioral Statistics*. 30:261-293.

17. Knapp G, Hartung J (2003): Improved tests for a random effects meta‐regression with a single covariate. *Statistics in medicine*. 22:2693-2710.

18. Higgins JP, Thompson SG (2002): Quantifying heterogeneity in a meta-analysis. *Stat Med*. 21:1539-1558.

19. Egger M, Smith GD, Schneider M, Minder C (1997): Bias in meta-analysis detected by a simple, graphical test. *bmj*. 315:629-634.

20. Sterne JA, Sutton AJ, Ioannidis JP, Terrin N, Jones DR, Lau J, et al. (2011): Recommendations for examining and interpreting funnel plot asymmetry in meta-analyses of randomised controlled trials. *Bmj*. 343:d4002.

21. National Heart Lung and Blood Institute (NHLBI) (2021): Study Quality Assessment Tools.

22. Aftanas LI, A. FE, S. AM, A. BD, V. RM, G. SG, et al. (2023): The habenular volume and PDE7A allelic polymorphism in major depressive disorder: preliminary findings. *The World Journal of Biological Psychiatry*. 24:223-232.

23. Etienne J, Boutigny A, Minh Ngoc Thien KTD, Ducreux D, Deflesselle E, Chappell K, et al. (2023): Habenular volume in depressed patients. *Psychiatry Clin Neurosci*. 77:191-192.

24. Furman DJ, Gotlib IH (2016): Habenula responses to potential and actual loss in major depression: preliminary evidence for lateralized dysfunction. *Soc Cogn Affect Neurosci*. 11:843-851.

25. Hou L, Bian B, Luan S, Pan X, Li M, Xue H, et al. (2025): High-resolution structural magnetic resonance examination of the habenula in patients with first-episode depression: an exploratory radiomics diagnostic value analysis based on cluster analysis.

26. Kyuragi Y, Oishi N, Hatakoshi M, Hirano J, Noda T, Yoshihara Y, et al. (2024): Segmentation and Volume Estimation of the Habenula Using Deep Learning in Patients With Depression. *Biological Psychiatry Global Open Science*. 4:100314.

27. Luan Sx, Zhang L, Wang R, Zhao H, Liu C (2019): A resting‐state study of volumetric and functional connectivity of the habenular nucleus in treatment‐resistant depression patients. *Brain and Behavior*. 9:e01229.

28. Schafer M, Kim JW, Joseph J, Xu J, Frangou S, Doucet GE (2018): Imaging Habenula Volume in Schizophrenia and Bipolar Disorder. *Front Psychiatry*. 9:456.

29. Amiri S, Arbabi M, Rahimi M, Parvaresh-Rizi M, Mirbagheri MM (2023): Effective connectivity between deep brain stimulation targets in individuals with treatment-resistant depression. *Brain Commun*. 5:fcad256.

30. Carceller-Sindreu M, de Diego-Adeliño J, Serra-Blasco M, Vives-Gilabert Y, Martí, n-Blanco A, et al. (2015): Volumetric MRI study of the habenula in first episode, recurrent and chronic major depression. *European Neuropsychopharmacology*. 25:2015-2021.

31. Aftanas L, Lipina T, Rezakova M, Filimonova E, Pustovoyt S, Novozhilova N, et al. (2020): Association of Habenula Volume With Anhedonia, Pathological Rumination, and EEG Response to Motivationally Relevant Stimuli in Depression. *Biological Psychiatry*. 87:S366.

32. Gao J, Li Y, Wei Q, Li X, Wang K, Tian Y, Wang J (2021): Habenula and left angular gyrus circuit contributes to response of electroconvulsive therapy in major depressive disorder. *Brain Imaging Behav*. 15:2246-2253.

33. Sartorius A, Demirakca T, Böhringer A, Clemm von Hohenberg C, Aksay SS, Bumb JM, et al. (2016): Electroconvulsive therapy increases temporal gray matter volume and cortical thickness. *European Neuropsychopharmacology*. 26:506-517.

34. Etienne J, Boutigny A, David DJ, Deflesselle E, Gressier F, Becquemont L, et al. (2024): Habenular volume changes after venlafaxine treatment in patients with major depression. *Psychiatry Clin Neurosci*. 78:468-472.

35. Ely BA (2019): Function and Anatomy of the Human Habenula Circuit [Ph.D.]. United States -- New York: Icahn School of Medicine at Mount Sinai.

36. Kumar P, Goer F, Murray L, Dillon DG, Beltzer ML, Cohen AL, et al. (2018): Impaired reward prediction error encoding and striatal-midbrain connectivity in depression. *Neuropsychopharmacology*. 43:1581-1588.

37. Savitz JB, Bonne O, Nugent AC, Vythilingam M, Bogers W, Charney DS, Drevets WC (2011): Habenula volume in post-traumatic stress disorder measured with high-resolution MRI. *Biol Mood Anxiety Disord*. 1:7.

38. Sartorius A, Demirakca T, Böhringer A, Clemm von Hohenberg C, Aksay SS, Bumb JM, et al. (2019): Electroconvulsive therapy induced gray matter increase is not necessarily correlated with clinical data in depressed patients. *Brain Stimul*. 12:335-343.

39. Dai Q, Kyuragi Y, Zakia H, Oishi N, Yao L, Zhang Z, et al. (2024): Psychological resilience is positively correlated with Habenula volume. *Journal of Affective Disorders*. 365:178-184.

40. Samanci B, Tan S, Michielse S, Kuijf ML, Temel Y (2024): Habenula volume change in Parkinson's disease: A 7T MRI study. *Brain Res Bull*. 215:111002.

41. Elias GJB, Germann J, Loh A, Boutet A, Pancholi A, Beyn ME, et al. (2022): Habenular Involvement in Response to Subcallosal Cingulate Deep Brain Stimulation for Depression. *Front Psychiatry*. 13:810777.

42. Johnston BA, Steele JD, Tolomeo S, Christmas D, Matthews K (2015): Structural MRI-Based Predictions in Patients with Treatment-Refractory Depression (TRD). *PLoS One*. 10:e0132958.

43. Jung JY, Cho SE, Kim N, Kang CK, Kang SG (2022): Decreased resting-state functional connectivity of the habenula-cerebellar in a major depressive disorder. *Front Psychiatry*. 13:925823.

44. Anand A, Nakamura K, Spielberg JM, Cha J, Karne H, Hu B (2020): Integrative analysis of lithium treatment associated effects on brain structure and peripheral gene expression reveals novel molecular insights into mechanism of action. *Translational psychiatry*. 10:103.

45. Diego-Adeliño J, Carceller M, Serra-Blasco M, Vives-Gilabert Y, Gómez-Ansón B, Dolors P, et al. (2013): *P.4.023 Habenular nuclei in different phases of major depressive disorder: a magnetic resonance imaging volumetric study*.

46. Salas RG, Savannah; Poblete, Guillermo; Nguyen, Tien; Madan, Alok; Fowler, Christopher (2017): A Novel Approach to Link Genetics and Human Brain Imaging Identifies Parahippocampal Gyrus to Habenula Resting State Functional Connectivity as Altered in Suicidality. *American College of Neuropsychopharmacology (ACPN) 56th Annual Meeting*. Palm Springs, CA, USA: American College of Neuropsychopharmacology, pp Poster Session I.
